# Hemoglobin state-flux: A finite-state model representation of the hemoglobin signal for evaluation of the resting state and the influence of disease

**DOI:** 10.1371/journal.pone.0198210

**Published:** 2018-06-08

**Authors:** Randall L. Barbour, Harry L. Graber, San-Lian S. Barbour

**Affiliations:** 1 Department of Pathology, SUNY Downstate Medical Center, Brooklyn, NY, United States of America; 2 Photon Migration Technologies Corp., Brooklyn, NY, United States of America; CNR, ITALY

## Abstract

**Summary:**

In this report we introduce a weak-model approach for examination of the intrinsic time-varying properties of the hemoglobin signal, with the aim of advancing the application of functional near infrared spectroscopy (fNIRS) for the detection of breast cancer, among other potential uses. The developed methodology integrates concepts from stochastic network theory with known modulatory features of the vascular bed, and in doing so provides access to a previously unrecognized dense feature space that is shown to have promising diagnostic potential. Notable features of the methodology include access to this information solely from measures acquired in the resting state, and analysis of these by treating the various components of the hemoglobin (Hb) signal as a co-varying interacting system.

**Approach:**

The principal data-transform kernel projects Hb state-space trajectories onto a coordinate system that constitutes a finite-state representation of covariations among the principal elements of the Hb signal (*i*.*e*., its oxygenated (ΔoxyHb) and deoxygenated (ΔdeoxyHb) forms and the associated dependent quantities: total hemoglobin (ΔtotalHb = ΔoxyHb + ΔdeoxyHb), hemoglobin oxygen saturation (ΔHbO_2_Sat = 100Δ(oxyHb/totalHb)), and tissue-hemoglobin oxygen exchange (ΔHbO_2_Exc = ΔdeoxyHb—ΔoxyHb)). The resulting ten-state representation treats the evolution of this signal as a one-space, spatiotemporal network that undergoes transitions from one state to another. States of the network are defined by the algebraic signs of the amplitudes of the time-varying components of the Hb signal relative to their temporal mean values. This assignment produces several classes of coefficient arrays, most with a dimension of 10×10.

**Biological motivation:**

Motivating our approach is the understanding that effector mechanisms that modulate blood delivery to tissue operate on macroscopic scales, in a spatially and temporally varying manner. Also recognized is that this behavior is sensitive to nonlinear actions of these effectors, which include the binding properties of hemoglobin. Accessible phenomenology includes measures of the kinetics and probabilities of network dynamics, which we treat as surrogates for the actions of feedback mechanisms that modulate tissue-vascular coupling.

**Findings:**

Qualitative and quantitative features of this space, and their potential to serve as markers of disease, have been explored by examining continuous-wave fNIRS 3D tomographic time series obtained from the breasts of women who do and do not have breast cancer. Inspection of the coefficient arrays reveals that they are governed predominantly by first-order rate processes, and that each array class exhibits preferred structure that is mainly independent of the others. Discussed are strategies that may serve to extend evaluation of the accessible feature space and how the character of this information holds potential for development of novel clinical and preclinical uses.

## Introduction

Breast cancer is a diverse disease that produces a number of prominent phenotypic behaviors that distinguish it from others. Hallmarks of cancer include enhanced angiogenesis, evasion of immune surveillance, presence of a sustained inflammatory response, enhanced tissue stiffness, resistance to cell death, and activation of invasion and metastasis, among others [1,2].

One avenue of development aimed at exploiting these characteristics is the use of imaging methods. A focus of these has been detection of contrast features linked to tumor angiogenesis [3]. For example, the understanding that tumor vessels are malformed and leaky has prompted the use of MR contrast imaging [4]. Also, appreciation that tumors often have increased vascular density has motivated use of near infrared spectroscopy (NIRS) as a tool for measuring disease-dependent features linked to the hemoglobin (Hb) signal. Extension of the NIRS technique in support of imaging has demonstrated that reliable detection of larger tumors appears feasible [5,6], while early disease detection is problematic owing to image blurring caused by scattering. Although this limitation is inherent to the method, other strategies for extending the method’s utility have been considered. One approach has been to use it as a confirmatory technique, in the hope that identification of additional features may serve to limit the number of false positive findings [7]. Prior knowledge also has been employed [8], as, among other things, a basis for monitoring the response to neoadjuvant chemotherapy [9].

A different approach, advanced by our group, has the aim of extending detection of tumor contrast features into the time domain, in recognition that either intrinsic or induced behaviors may provide for added disease detection and discrimination [10,11]. Reports exploring this strategy have considered implementation of Valsalva maneuvers [12,13] or mechanical force applied to the breast [14], to disturb blood flow to affected tissue in a discernible way. That disease discriminatory features may arise from these maneuvers follows from the aforementioned disturbances in tumor vessel morphology, which lead to increased interstitial pressures and reduced flow [15]. While examination of the full potential of these maneuvers is incomplete, reliance on evoked-response protocols seems likely to add to the operational complexity of the technique.

Recently we have reported evidence that detection of breast cancer may be feasible, even for small tumors, from measures obtained under resting-state conditions [16]. Compared to the mentioned maneuvers, this approach lessens concerns of patient compliance, while also likely reducing instrumentation requirements. Still, experience in exploring resting-state behaviors of the Hb signal from intact tissue with NIRS is limited. In the case of neuroimaging applications, the understanding that neural structures form a functional network has prompted adoption of signal recognition methods that are sensitive to the presence of coordinated behaviors [17]. However, because peripheral tissues, including the breast, lack structural elements equivalent to those enabling the brain to exhibit such behaviors, extension of these methods to other tissue types does not appear well justified.

The general problem of recognizing features from measures of time-varying behaviors is one that spans many fields of study. In the case of natural systems, observed behaviors are often not simply a consequence of one class of time-varying phenomenology, but rather arise from interactions among the principal factors that determine system behavior. A corollary of this is that while elementary features (*e*.*g*., frequency structure) of the individual components that define state behaviors can be considered, the full extent of system behavior is not observable from exploration of any one component. This understanding has important consequences for attempts at appreciating principal behaviors tied to the Hb signal. In particular, we recognize that most reports on NIRS measures of tissue, whether acquired in a resting state or under other conditions, characterize behaviors based on evaluation of individual Hb-signal elements and not as a system of interacting components.

In a previous report we outlined a basic methodology that recognized this feature, but it lacked a formal mathematical framework [18]. Here our aim has been to expand on this by implementing a modeling scheme that is motivated from understandings taken from the field of stochastic network theory. As will be shown, while our principal focus has been to achieve a useful methodology for evaluation of breast cancer, the developed approach appears sufficiently general to find uses in other applications as well.

### Rationale for model development

Investigation of time-varying behaviors of naturally occurring phenomena is often pursued by adopting one of two different modeling strategies. Strong models are descriptions that relate specific behaviors among variables in a well-defined manner. They explore data from the premise that specific relationships are assignable and are valid under the conditions of measurement. Weak models, on the other hand, make no attempt to invoke specific relationships, but instead seek to appreciate dependences among features in a probabilistic sense. Each approach has its value and tradeoffs.

Strong models support specific predictions at the cost of ignoring other phenomenology (*i*.*e*., any that is not explicitly included in the model), and often with the goal of identifying non-observable behaviors. Weak models explore directly observable features or those that can be readily extracted by use of various data transforms. As a practical matter, experience shows that weak model formalisms can be applied over a larger range of experimental conditions than strong models, and is the preferred approach for clinical measures. Because the value of these often depends on how effectively disease can be distinguished from non-disease, key is to balance the experimental conditions against the analysis methodology in the hope that distinguishable features are expressed with sufficient fidelity for the applied sensing methodology to register this information. Assuming the latter condition is met, it is useful to employ a feature interrogation strategy that broadly supports inclusion of the observable phenomenology in ways that do not require detailed prior knowledge of system mechanisms. Additionally, while it might seem evident that the selected sensing technology should meet the conditions thus put forth, what is not obvious is that disease-discriminating phenomenology has been or even could be sampled in a manner that optimally balances its expression under the conditions of measurement and its recoverability by the applied analysis method. It follows that, in principle, a well-considered weak-model approach is one that weighs the intersections among sensing conditions, feature expression, and feature recovery, in relation to the influence that disease is likely to have on observable features.

While this composite can take many forms, we are drawn to the properties of a finite Markov chain (FMC) as a simplified approximation of the behaviors that the co-varying elements of the Hb-signal can be expected to exhibit. A factor motivating this choice is our recognition that, apart from its oxygen delivery function, Hb also plays a key role in the trafficking of metabolically generated gases. Among these are carbon dioxide and nitric oxide (NO), both of which are known to have allosteric effects on the affinity of oxygen binding to hemoglobin [19,20] and also influence the reactivity of the microvascular bed [21,22]. Having a nonlinear influence, and similar to behaviors of other nonlinear systems, these effects can act to drive system behavior from one preferred state to another. Also favoring a weak-model formalism is the expectation that under resting-state conditions the feedback mechanisms affecting the microvasculature act to maintain a roughly steady-state condition, and thus the associated transition rates among elements of the nodal network are likely to reflect these influences. As is reported here, the outcome of this composite is a methodology that is simply applied, with good subject compliance, and that avails access to a previously unrecognized dense feature space whose properties appear favorable for disease detection and characterization.

## Methods

### Relationships among co-varying elements of the Hb signal

**Fig 1:**
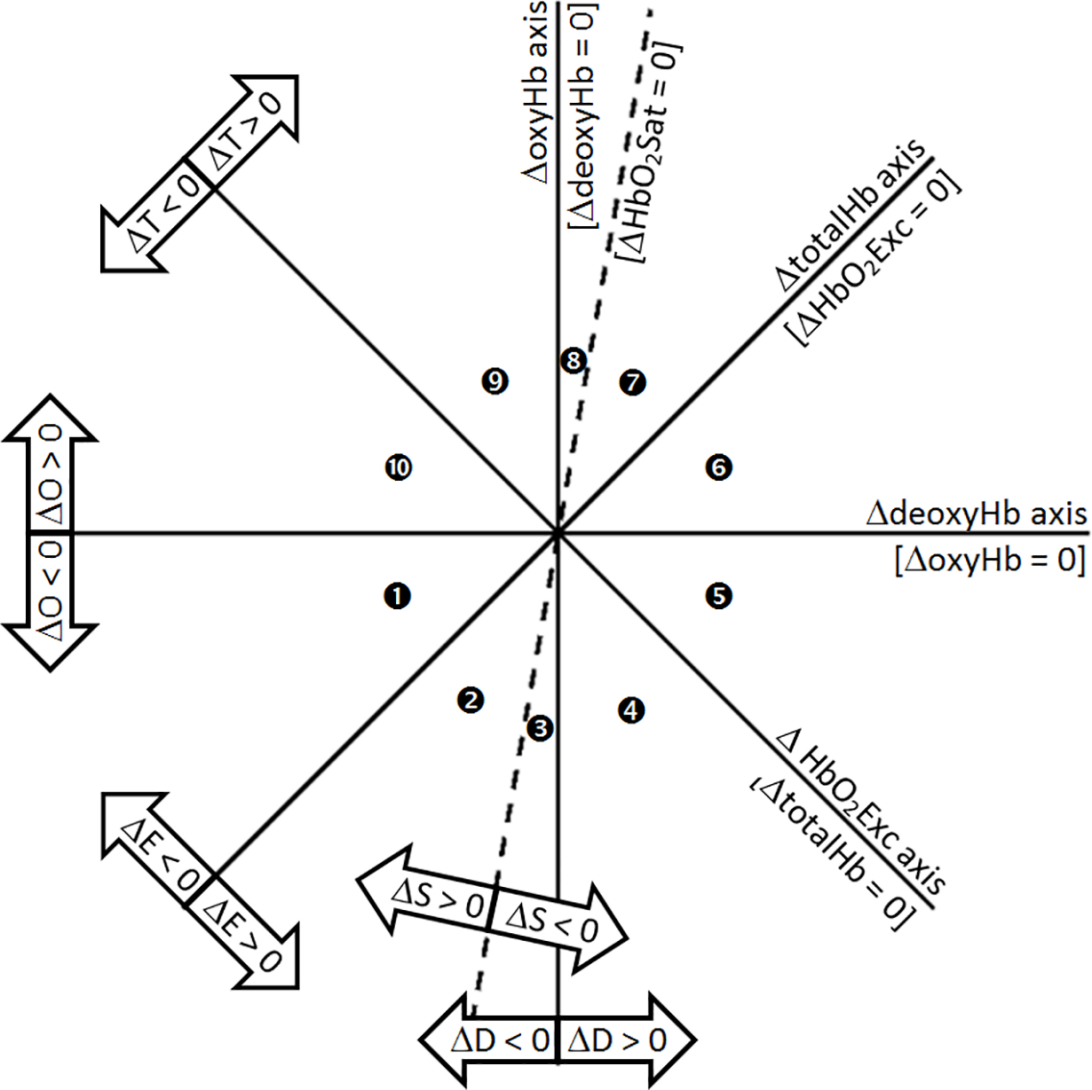
Coordinate axes defining the Hb states. Regions in (ΔdeoxyHb, ΔoxyHb) space that correspond to increasing or decreasing values, with respect to the temporal mean, for each commonly considered component of the Hb signal. Numbered circles show the Hb state assigned to each of the ten sectors defined by the five lines that intersect at the origin.

Fig 1 illustrates the coordinate system applied to derive co-varying features of the Hb-signal that are described by our weak-model formalism and applied to measures acquired under the resting state. Assigned to the principal Cartesian axes are the simultaneously measured values of relative changes in the oxygenated (ΔoxyHb) and deoxygenated (ΔdeoxyHb) forms of hemoglobin. Also represented are concurrent relative changes in the three computed quantities of the Hb signal comprising total Hb, (ΔtotalHb = ΔdeoxyHb + ΔoxyHb), Hb oxygen saturation (ΔHbO_2_Sat = 100Δ(oxyHb/totalHb)) and tissue-Hb oxygen exchange, ΔHbO_2_Exc = ΔdeoxyHb—ΔoxyHb). It can be shown that the correct inclination angle for the ΔHbO_2_Sat demarcation line with respect to the ΔdeoxyHb axis is tan^-1^(*S*_0_/(100 –*S*_0_)), where *S*_0_ is the temporal mean value (in percentage) of HbO_2_Sat. The inclination angle depicted in Fig 1 corresponds to *S*_0_ = 85% [6,23]. [see S1 Appendix for more compete derivation and explanation of the assigned axes.]

Within this representation, plots of the time-varying behavior of the Hb signal trace out a trajectory in state-space. As described below, we have elected to model this behavior not as a continuous variable, but rather as a set of ten interacting finite states that are represented by the spaces between the identified axes. The trajectory data can originate from primary time-series measures of the Hb signal or from subsequent transformations that produce tomographic images. For simplicity, in this representation each state is defined with respect to the algebraic sign of each element of the hemoglobin signal relative to its temporal mean value, and the figure is annotated to reflect these definitions. Also, to provide a more complete characterization of the co-varying states, hereafter referred as Hb states, in Table 1 we identify additional mathematical relations that are implicit in each state’s algebraic-sign permutation. For example, State 10 combines ΔoxyHb > 0 with ΔdeoxyHb and ΔtotalHb both < 0. A negative ΔtotalHb value is possible only if the ΔdeoxyHb concentration is below its temporal mean value by an amount greater than that by which ΔoxyHb exceeds its own mean concentration, and this fact is indicated in the right-most column of Table 1. Thus the Hb state definitions, by incorporating information on all five Hb components, are based on the magnitudes of ΔdeoxyHb and ΔoxyHb in addition to their algebraic signs.

**Table 1:** Hb states defined by the algebraic signs of Hb signal components relative to their respective temporal mean values.

State	Algebraic sign of Component^(a^Δ*O*Δ*D*Δ*T*Δ*E*Δ*S*	Associated Mathematical Relations^(b^
1	- - - - +	Δ*E*: |Δ*O*| < |Δ*D*| Δ*S*: |Δ*O*|/*O*_0_ < |Δ*T*|/*T*_0_
2	- - - + +	Δ*E*: |Δ*O*| > |Δ*D*| Δ*S*: |Δ*O*|/*O*_0_ < |Δ*T*|/*T*_0_
3	- - - + -	Δ*E*: |Δ*O*| > |Δ*D*| Δ*S*: |Δ*O*|/*O*_0_ > |Δ*T*|/*T*_0_
4	- + - + -	Δ*S*: |Δ*O*|/*O*_0_ > |Δ*T*|/*T*_0_ Δ*T*: |Δ*O*| > Δ*D*
5	- + + + -	Δ*T*: |Δ*O*| < Δ*D*
6	+ + + + -	Δ*E*: Δ*O* < Δ*D* Δ*S*: Δ*O*/*O*_0_ < Δ*T*/*T*_0_
7	+ + + - -	Δ*E*: Δ*O* > Δ*D* Δ*S*: Δ*O*/*O*_0_ < Δ*T*/*T*_0_
8	+ + + - +	Δ*E*: Δ*O* > Δ*D* Δ*S*: Δ*O*/*O*_0_ > Δ*T*/*T*_0_
9	+ - + - +	Δ*S*: Δ*O*/*O*_0_ > Δ*T*/*T*_0_ Δ*T*: Δ*O* > |Δ*D*|
10	+ - - - +	Δ*T*: Δ*O* < |Δ*D*|

a) Letters with ‘0’ subscripts denote temporal mean values; letters preceded by ‘Δ’ denote differences with respect to the temporal mean value. ‘*O*_0_’ = oxyHb, ‘Δ*O*’ = ΔoxyHb; ‘Δ*D*’ = ΔdeoxyHb; ‘*T*_0_’ = totalHb, ‘Δ*T*’ = ΔtotalHb; ‘Δ*E*’ = ΔHbO_2_Exc; ‘Δ*S*’ = ΔHbO_2_Sat.

b) The inequalities in this column are implicit in the state definitions. That is, when ‘Δ*D*’ and ‘Δ*O*’ have the same algebraic sign, the sign of ‘Δ*E*’ determines whether Δ*D* or Δ*O* has the larger magnitude; when Δ*D* and Δ*O* have opposite signs, the sign of ‘Δ*T*’ determines whether Δ*D* or Δ*O* has the larger magnitude; and when Δ*O* and Δ*T* have the same sign, the sign of ‘Δ*S*’ determines whether Δ*O*/*O*_0_ or Δ*T*/*T*_0_ has the larger magnitude.

The identified coordinate system is an extension of the 6-state co-varying system originally considered by our group [18,24]. It also represents an extension of subsequent reports that recognized added information accessible from a co-varying representation of the hemoglobin components [25,26].

### Quantification of inter-state Hb-transition coefficients

Every image voxel in a reconstructed image time series occupies exactly one of the ten Hb states at each measurement time frame. Using *v* and *t* for the space and time variables, respectively, and Δ*t* for the reciprocal of the data sampling rate, *s*(*v*,*t*) denotes the state of the *v*^th^ voxel in the *t*^th^ time frame and *s*(*v*,*t*+*n*Δ*t*) is the state of the same voxel *n* time frames later. The approach we take counts only the direct transitions from one state into a different one [as described in S2 Appendix], and excludes all instances of *s*(*v*,*t*)→*s*(*v*,*t*+*n*Δ*t*) that have intervening transitions within the *n*-time-frame lag. Thus, we count all one-step transitions of a specified type, irrespective of the time interval over which those transitions occur. This has the effect of defining transitions based on a variable dwell time [See S3 Appendix for a more detailed description of the transition-assignment methodology]. Also excluded from the applied state definitions is any consideration of distance from the origin of the (ΔdeoxyHb, ΔoxyHb) coordinate system, even though we recognize the possibility that, for example, the portion of the State-1 sector (Fig 1) preferentially transitioned into could be a function of which of the other states (and their corresponding portion) was the predecessor. This postulated history dependence would distinguish the network dynamics from those of a true FMC.

#### Computation of state transition probability

At the start of a transition-probability computation, a 10×10×*N* array of integer variables, where *N* is the largest value of the time lag that will be considered (in practice it was found that *N* = 60 is sufficient for detection of all transitions), is initialized to all zeros. Each element of the three-dimensional array will accumulate a count of the number of transitions of a given type: position in the first dimension (“row”) encodes the Hb states that voxels transition into, in the second dimension (“column”) the state that they transition from, and in the third dimension (“layer”) the time lag for the transitions.

Starting with *v* = *t* = *n* = 1, the values of *s*(*v*,*t*) and *s*(*v*,*t*+*n*Δ*t*) are compared. If *s*(*v*,*t*+*n*Δ*t*) = *s*(*v*,*t*), *n* is incremented by 1 and the comparison is repeated; but if *s*(*v*,*t*+*n*Δ*t*) ≠ *s*(*v*,*t*), the value in the [*s*(*v*,*t*+*n*Δ*t*),*s*(*v*,*t*),*n*]^th^ element of the accumulator array is increased by 1. Each time the inequality criterion is met (thereby indicating that a transition to a different state has occurred), the current value of *t* is increased to *t*+*n*Δ*t* (*i*.*e*., the previous post-transition time frame becomes the new pre-transition time frame), and the procedure is repeated in order to identify the next transition. This process of counting transitions and incrementing *t* continues until the end of the time series is reached, at which point the value of *v* is incremented, *t* and *n* are reset to 1, and the preceding sequence of operations is repeated until the maximum values of *v* and *t* are reached.

We use the symbol **A** to denote the accumulator array, and *f* (for “final state”) and *i* (“initial state”) for the row and column indices, respectively. For *f* ≠ *i*, (the *f* = *i* elements are superfluous in this analysis) the sum $\sum_{n=1}^{N}A_{fi}\left(n\right)$ is the total number of transitions directly from state *i* to state *f*, irrespective of the number of intervening time frames. The sum of accumulated values in every layer of the array is proportional to the subject-specific time-series duration and voxel count. Accordingly, to allow for computation of average transition matrices across subjects, we normalize the sum of transition counts to 100, including only the 90 *f* ≠ *i* elements in the normalization. Thus

$$P_{fi}=\frac{100\sum_{n=1}^{N}A_{fi}\left(n\right)}{\sum_{f=1}^{10}\sum_{i=1}^{10}\left(1-\delta_{fi}\right)\sum_{n=1}^{N}A_{fi}\left(n\right)},$$ (1)

where *δ*_*fi*_ is the Kronecker delta.

#### Computation of state transition rate

The approach taken here also allows for extraction of a mean time-lag value for each transition type:

$$\tau_{fi}=\frac{\sum_{n=1}^{N}n\cdot A_{fi}\left(n\right)}{\sum_{n=1}^{N}A_{fi}\left(n\right)},$$ (2)

For *f* ≠ *i*, the computed *τ*_*fi*_ value is the average number of time frames associated with transitions from state *i* into state *f*. The mean time can be transformed to an equivalent rate constant for *i*→*f* transitions, where

$$k_{fi}=\frac{1}{\tau_{fi}}.$$ (3)

In Eq (3), *k*_*fi*_ has units of (time frame)^-1^ which is easily converted to seconds^-1^ by multiplying by the data sampling rate.

### Quantification of transition-linked hemoglobin-concentration and -saturation changes

A modification to the accumulation scheme allows us to determine amplitude-sensitive measures that are equivalent to the average amounts by which levels of the five components of the Hb signal change during each of the 90 *i*→*f* (*f* ≠ *i*) transition types. Thus, although the state definitions *per se* are independent of component amplitudes, and hence of distance from the origin in Fig 1, measures defined here are sensitive to this feature. In place of the single previously considered accumulator array, we now define six such arrays,

$$A_{TC},\;A_{D},\;A_{E},\;A_{O},\;A_{S},\;A_{T},$$ (4)

each having the same dimensionality and size as the accumulator considered in the preceding section and each initialized to all-zeros, and where the subscripts denote: *TC* = transition count, *D* = ΔdeoxyHb, *E* = ΔHbO_2_Exc, *O* = ΔoxyHb, *S* = ΔHbO_2_Sat, *T* = ΔtotalHb. For each transition, values stored in appropriate elements of the accumulators are incremented as follows:

$$\begin{array}{l} A_{TC}\left(s\left(v,t+n\Delta t\right),s\left(v,t\right),n\right)\to A_{TC}\left(s\left(v,t+n\Delta t\right),s\left(v,t\right),n\right)+1,\\ A_{D}\left(s\left(v,t+n\Delta t\right),s\left(v,t\right),n\right)\to A_{D}\left(s\left(v,t+n\Delta t\right),s\left(v,t\right),n\right)\\ \;+\mathrm{deoxyHb}\left(v,t+n\Delta t\right)-\mathrm{deoxyHb}\left(v,t\right),\\ A_{E}\left(s\left(v,t+n\Delta t\right),s\left(v,t\right),n\right)\to A_{E}\left(s\left(v,t+n\Delta t\right),s\left(v,t\right),n\right)\\ \;+{\mathrm{HbO}}_{2}\mathrm{Exc}\left(v,t+n\Delta t\right)-{\mathrm{HbO}}_{2}\mathrm{Exc}\left(v,t\right),\\ A_{O}\left(s\left(v,t+n\Delta t\right),s\left(v,t\right),n\right)\to A_{O}\left(s\left(v,t+n\Delta t\right),s\left(v,t\right),n\right)\\ \;+\mathrm{oxyHb}\left(v,t+n\Delta t\right)-\mathrm{oxyHb}\left(v,t\right),\\ A_{S}\left(s\left(v,t+n\Delta t\right),s\left(v,t\right),n\right)\to A_{S}\left(s\left(v,t+n\Delta t\right),s\left(v,t\right),n\right)\\ \;+{\mathrm{HbO}}_{2}\mathrm{Sat}\left(v,t+n\Delta t\right)-{\mathrm{HbO}}_{2}\mathrm{Sat}\left(v,t\right),\\ A_{T}\left(s\left(v,t+n\Delta t\right),s\left(v,t\right),n\right)\to A_{T}\left(s\left(v,t+n\Delta t\right),s\left(v,t\right),n\right)\\ \;+\mathrm{totalHb}\left(v,t+n\Delta t\right)-\mathrm{totalHb}\left(v,t\right). \end{array}$$ (5)

#### Transition flux computation

The transition flux for a given hemodynamic-signal component is equal to the average change in concentration or saturation, per transition of a specified type. We refer to these quantities as representative of a ‘flux’, in a similar manner to ion fluxes that occur across biological membranes, or to fluxes of material through the chemical intermediates in a metabolic pathway. Mathematically, the flux for transitions from state *i* to state *f* is equal to

$$\phi_{X}\left(f,i\right)=\frac{\sum_{n=1}^{N}A_{X}\left(f,i,n\right)}{\sum_{n=1}^{N}A_{TC}\left(f,i,n\right)},$$ (6)

where the generic symbol *X* is used to denote any of the five hemoglobin-signal components *D*, *E*, *O*, *S* or *T*. Unlike the single arrays corresponding to the magnitude-independent coefficients, here we obtain five sets of 10×10 arrays, one for each component of the Hb signal.

As will be shown in Results, coefficient maps corresponding to component flux, and state transition rate and probability, reveal distinct contrast features which suggest these can be treated as mainly independent quantities. This leads to the understanding that it may also be useful to examine ‘weighted’ quantities as defined by various combinations of coefficient values.

#### Computation of weighted transition fluxes

A parameter combination that includes transition flux (*ϕ*) as one of the factors can be conceptualized as a ‘weighted flux’, with the remaining factor (*k* or *P*) constituting the weighting term. The *P*_*fi*_*ϕ*_*X*_(*f*,*i*) product for a given hemodynamic-signal component is a quantity that we call the transition mass [*m*_*X*_(*f*,*i*)] and is equal to the average concentration or saturation change that occurs during the time required for a set number of transitions to happen. Proceeding from Eqs (1) and (6), we see that the mass for transitions from state *i* to state *f*, for signal component *X*, is

$$\begin{array}{l} m_{X}\left(f,i\right)=P_{fi}\phi_{X}\left(f,i\right)=\frac{100\sum_{n=1}^{N}A_{TC}\left(f,i,n\right)}{\sum_{f=1}^{10}\sum_{i=1}^{10}\left(1-\delta_{fi}\right)\sum_{n=1}^{N}A_{TC}\left(f,i,n\right)}\frac{\sum_{n=1}^{N}A_{X}\left(f,i,n\right)}{\sum_{n=1}^{N}A_{TC}\left(f,i,n\right)}\\ \;=100\frac{\sum_{n=1}^{N}A_{X}\left(f,i,n\right)}{\sum_{f=1}^{10}\sum_{i=1}^{10}\left(1-\delta_{fi}\right)\sum_{n=1}^{N}A_{TC}\left(f,i,n\right)}. \end{array}$$ (7)

The quantity *m*_*X*_(*f*,*i*) defined in Eq (7) can be conceptualized as the average quantity of material that is transferred from State *i* to State *f* whenever 100 transitions overall (*i*.*e*., including all types) occur. Multiplying either *ϕ*_*X*_(*f*,*i*) or *m*_*X*_(*f*,*i*) by *k*_*fi*_ produces a quantity in which the concentration or saturation changes are given per unit time rather than on a per-transition basis. The corresponding mathematical formulations are

$$k_{fi}\phi_{X}\left(f,i\right)=\frac{\sum_{n=1}^{N}A_{TC}\left(f,i,n\right)}{\sum_{n=1}^{N}n\cdot A_{TC}\left(f,i,n\right)}\frac{\sum_{n=1}^{N}A_{X}\left(f,i,n\right)}{\sum_{n=1}^{N}A_{TC}\left(f,i,n\right)}=\frac{\sum_{n=1}^{N}A_{X}\left(f,i,n\right)}{\sum_{n=1}^{N}n\cdot A_{TC}\left(f,i,n\right)}$$ (8)

and

$$k_{fi}m_{X}\left(f,i\right)=100\frac{\sum_{n=1}^{N}A_{TC}\left(f,i,n\right)}{\sum_{n=1}^{N}n\cdot A_{TC}\left(f,i,n\right)}\cdot \frac{\sum_{n=1}^{N}A_{X}\left(f,i,n\right)}{\sum_{f=1}^{10}\sum_{i=1}^{10}\left(1-\delta_{fi}\right)\sum_{n=1}^{N}A_{TC}\left(f,i,n\right)}.$$ (9)

To distinguish the influence of this rescaling process, we refer to unweighted coefficients [*i*.*e*., Eq (6)] as intrinsic coefficient values, while those listed in Eqs (7)–(9) are called weighted coefficient values.

For completeness, we acknowledge without further consideration still another class of coefficients, which is representative of asymmetry between pairs of transitions A→B and B→A. This recognizes that the rate, probability and flux coefficients (and the associated weighted values) for a given transition need not have the same values as those for the reverse transition.

### Influence of algebraic sign of Hb-component on transition flux amplitude

In Eq (5), the increments for all of the **A** arrays except **A**_*TC*_ are defined as a difference between the concentration or saturation values at two identified time frames. From the way the Hb states are defined (see Fig 1, Table 1), it follows that for each component of the Hb signal some transition types produce only positive increments, others only negative ones, and others a mixture of positive and negative increments. This mixture produces different feature characteristics whose interpretation is aided by the following understandings.

**Fig 2:**
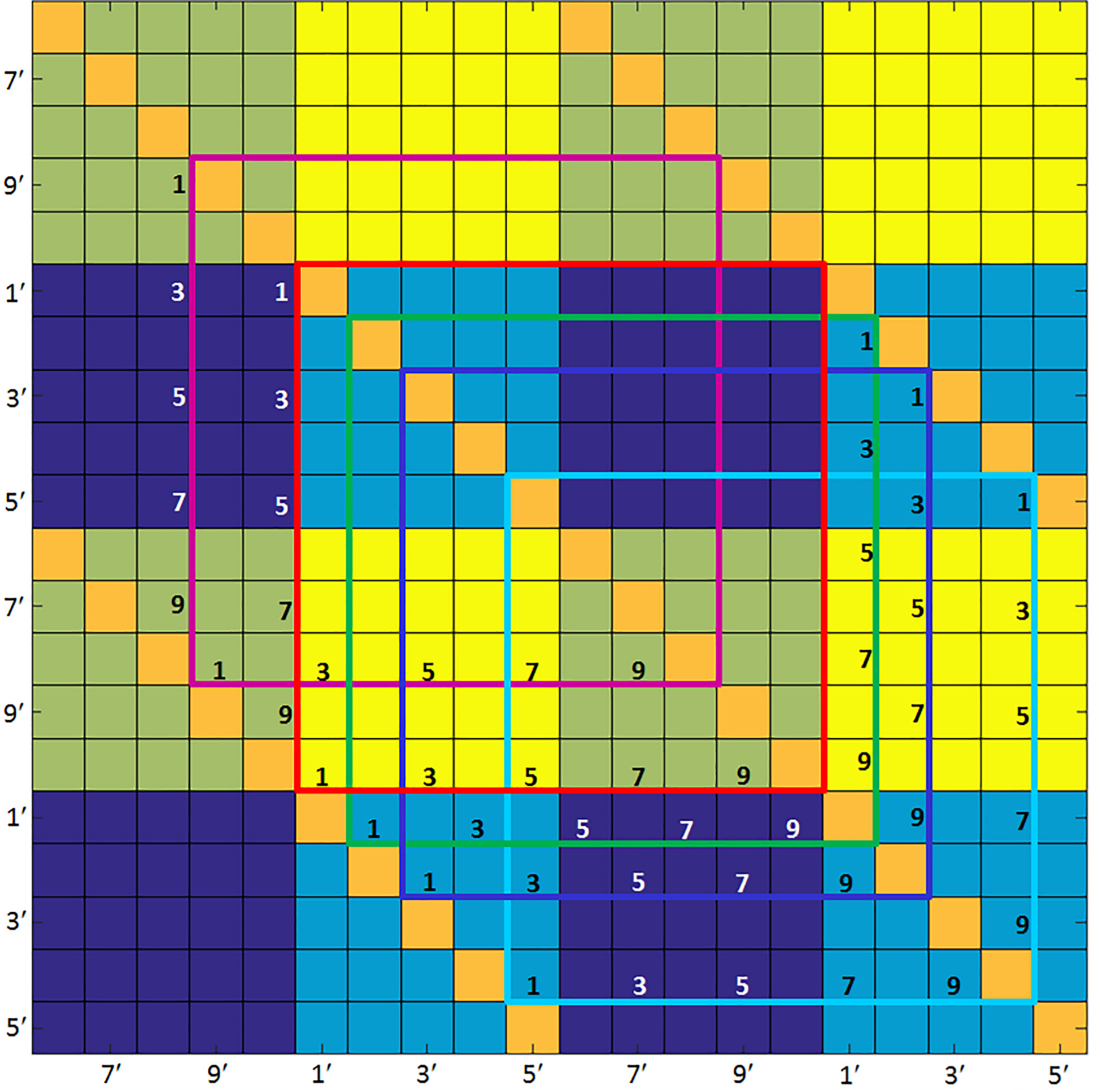
Relation between algebraic signs of pre- and post-transition ΔHb values, for each component of the Hb signal. Numbering along figure edges reflects the periodicity of the Hb-state sequence (see Fig 1). Numbers along edges of colored bounding boxes indicate the pre- and post-transition states (along the horizontal and vertical edges, respectively), for each component: red = ΔoxyHb, dark blue = ΔdeoxyHb, green = ΔtotalHb, light blue = ΔHbO_2_Exc, magenta = ΔHbO_2_Sat. Solid-block colors indicate: light blue = (-)→(-) transitions, dark blue = (+)→(-), olive = (+)→(+), yellow = (-)→(+).

The algebraic signs associated with different transition types are coded as colored regions in Fig 2. The dark blue color indicates transitions from a positive (*i*.*e*., greater than the temporal mean value) to a negative value, in which case the concentration or saturation must decrease; while yellow indicates transitions from negative to positive values, so that concentration or saturation must increase. The light-blue color corresponds to transitions from one negative value to another, in which case the Hb-component value will increase in some transitions and decrease in others; and the same is true for the olive-colored regions, which correspond to transitions from one positive value to another. The color-coded boundary squares in Fig 2 show which portions of the checkerboard pattern correspond to the algebraic-sign distributions for each Hb component. Note that the regions inside the colored boundary squares have the same row and column assignments. Thus the cell in the upper right corner of each boundary square corresponds to State-10 → State-1 transitions. As is shown in Results, there are features in the associated coefficient-flux maps that are spatially correlated with those in Fig 2.

Separate from these feature characteristics, it is helpful to appreciate the influence that changes in state-dependent flux amplitudes will have on the appearance of transition-parameter maps. For instance, transitions that must entail a change in algebraic sign will identify the net amplitude of the concentration or saturation change, but not the magnitude of the hemoglobin signal relative to its temporal mean value. In contrast, transitions in which the algebraic sign does not change will produce net values that constitute sums of cancelations. As a result, these net values may be either positive or negative, and the observed algebraic sign will indicate whether the pre- or post-transition value is farther from the temporal mean.

### Functional interpretation of Hb-state transitions

While the described mathematical treatments do not require assignment of a particular sequence to the state numbering system, we nevertheless have arranged these to reflect a gradient of successively greater degrees of metabolic demand in relation to blood supply with vascular compensation. Contrasting examples of this response are the transitions between states 5 and 10 and between 4 and 9. For instance, the 10→5 transition identifies a hyperemic response, but one that is insufficient to meet demand, leading the levels of ΔoxyHb and ΔHbO_2_Sat to fall below their temporal mean values while the levels of ΔHbO_2_Exc and ΔdeoxyHb rise. Conversely, the 4→9 transition principally reflects a similarly hyperemic response, but one that more than meets metabolic demand, leading ΔHbO_2_Exc values to fall while ΔoxyHb, ΔtotalHb, and ΔHbO_2_Sat levels rise. The associated reciprocal transitions reflect responses in compensation from hyperemia. It deserves mention that not specifically reflected in this representation are explicit sensitivities of these parameters to changes in blood flow separate from variations in tissue metabolic oxygen demand [27]. Nevertheless, as variations in these factors will affect the oxygen supply-demand balance, the finite-state formulation presented is expected to prove sensitive to factors influencing these quantities [see S4 Appendix for an expanded description of the physiological interpretation of state transitions].

### Evaluation of diagnostic potential of transition-associated parameters

Here we use the generic symbol *Y*_*fi*_ to denote any of the transition-related quantities—*P*_*fi*_, *k*_*fi*_, *ϕ*_*X*_(*f*,*i*), *m*_*X*_(*f*,*i*), or the related products *k*_*fi*_*ϕ*_*X*_(*f*,*i*), *k*_*fi*_*m*_*X*_(*f*,*i*)—that are subsequently evaluated as diagnostic indicators for breast cancer. For any transition type, each study subject has distinct values of *Y*_*fi*_ for the left and right breast (*i*.*e*., *Y*_*fi*_^*l*^ and *Y*_*fi*_^*r*^, respectively). To minimize contributions to inter-subject variability by factors unrelated to the presence of disease, we consider the relative percent differences between *Y*_*fi*_^*l*^ and *Y*_*fi*_^*r*^ as diagnostic-metric candidates.

In view of the demonstrational nature of the diagnostic-potential computations reported here, and the small number of subjects in the breast-cancer group (*N* = 18), we do not consider multiple-category or multiple-factor analyses such as would be appropriate for a large clinical trial. Instead, data for all breast-cancer subjects are combined to form one group. Because the 18 subjects with cancer comprise 12 with a tumor of the left breast and 6 who have it in the right, combining them necessitates performing an adjustment to minimize confounding of tumor effects with any intrinsic left-right asymmetries that may be present. Accordingly, the diagnostic metrics are generated in the following manner. For subjects known to have left-breast tumors (and for subjects in the non-cancer group):

$$Z_{fi}\left(j\right)=100\frac{Y_{fi}^{l}\left(j\right)-Y_{fi}^{r}\left(j\right)}{Y_{fi}^{r}\left(j\right)},$$ (10)

where *j* is the subject index. For subjects known to have right-breast tumors,

$$Z_{fi}\left(j\right)=100\frac{\left[Y_{fi}^{r}\left(j\right)-{\bar{Y_{fi}^{r}}}_{\mathrm{NC}}+{\bar{Y_{fi}^{l}}}_{\mathrm{NC}}\right]-\left[Y_{fi}^{l}\left(j\right)-{\bar{Y_{fi}^{l}}}_{\mathrm{NC}}+{\bar{Y_{fi}^{r}}}_{\mathrm{NC}}\right]}{Y_{fi}^{l}\left(j\right)-{\bar{Y_{fi}^{l}}}_{\mathrm{NC}}+{\bar{Y_{fi}^{r}}}_{\mathrm{NC}}},$$ (11)

where ${\bar{Y_{fi}^{l}}}_{\mathrm{NC}}$ and ${\bar{Y_{fi}^{r}}}_{\mathrm{NC}}$ denote quantities averaged over all subjects in the non-cancer group. The linear adjustment operations in Eq (11) have the effect of correcting the difference between parameter values in the tumor-bearing and unaffected breasts for left-right biases that are not related to the presence of disease. As such, they are based on a plausible assumption that the net inter-breast difference is, to first order, the sum of background-asymmetry and tumor-related contributions, and that the magnitude of the former is not itself strongly disease-dependent.

For each combination of transition-related parameter and transition type, the sets of *Z*_*fi*_ values for breast-cancer and non-cancer subjects were the input to a receiver operator characteristic (ROC) analysis [16], with the area-under-curve (AUC) parameter taken as our index of overall diagnostic accuracy [28].

### Diffuse optical tomography

Optical time series measures of the breast were acquired using a previously reported, custom-made, high density tomography system [14]. Notable capabilities of this unit include the capacity to examine both breasts simultaneously while, if desired, exposing the breast to concurrent defined viscoelastic deformations. Illumination was achieved by employing a dual-wavelength laser source (760, 830 nm) that is time-multiplexed to allow for simultaneous recording of light intensities from all elements of the sensing array. In all, each sensing head contains 64 dual-wavelength sources and 32 detector elements that are evenly divided within a two-stage arrangement.

### Subject preparation

System operation supports examination of the breast while the subject is comfortably seated. The top portion of the sensing array has a hinged arrangement enabling its adjustment to allow for controlled contact in an anatomically conforming manner. In all, sensing elements are arranged to support essentially a full circumferential measurement. Informed consent was obtained from all subjects prior to enrollment, in accordance with Project 267113–10 of the SUNY Downstate Medical Center Institutional Review Board, which approved this study. In all, data from 63 subjects were explored, 18 with confirmed breast cancer (6-right breast, 12-left breast, average tumor size ~2.7 cm, range 0.5–6 cm) and 45 non-cancer subjects, 23 of whom had evidence of various types of non-malignant pathologies in either breast. A more detailed description of enrolled subjects is given in [16]. It deserves mention that findings reported here involve the same subject groups as in [16] and exploration of the same data. Different are the methodologies applied for data analysis.

### Data collection

Following initial setup, automated system calibration was performed to identify optimal gain settings. Subsequently, resting-state measures were acquired for a period lasting ~5–10 minutes.

### Data preprocessing

Data were screened for evidence of degradation of signal quality caused by excessive signal attenuation by very large breasts or by poor skin-contact for very small breasts, using methods previously described [16]. To avoid introducing bilateral signal bias, channels excluded from one breast were also excluded from the other, resulting in symmetric data sets. Also, to avoid biases from undersampling, only measures from women that included at least 60% of all data channels were considered. Experience showed that operating limits are breasts having cup sizes varying from B to DD.

### 3D image reconstruction

Tomographic reconstructions were achieved by first normalizing measured signal intensities to the respective temporal mean values of each data channel, then solving a system of linear equations generated within a modified perturbation formulation that is based on solutions to the diffusion equation [29,30]. Computed wavelength-dependent absorption coefficient values were subsequently transformed to yield spatial maps of the time-varying independent (*i*.*e*., oxygenated and deoxygenated) components of the hemoglobin signal. Using the MATLAB ‘detrend’ command, a linear detrending operation was subsequently applied, across the time dimension, to the reconstructed ΔoxyHb and ΔdeoxyHb images, to remove any long-term drift (which, under the conditions of measurement, is more likely instrumental than of biological origin) that may be present in the data while retaining fluctuations on shorter time scales. The mathematical relationships given above (Methods, Section 1), were used to compute values of the dependent Hb components from the detrended ΔoxyHb and ΔdeoxyHb measures.

### Organization of reported findings

In the following section we present a representative finding whose gross features have aided in motivating adoption of the finite-state methodology described in Methods, and expand on group-level findings obtained from the different subject groups. The latter is divided into two sub-sections: findings from analysis of State-transitions, and those from Hb-dependent component transitions. Finally, we report selected findings from ROC analysis that supports evidence that the derived coefficients may serve as useful markers for disease.

## Results

**Fig 3:**
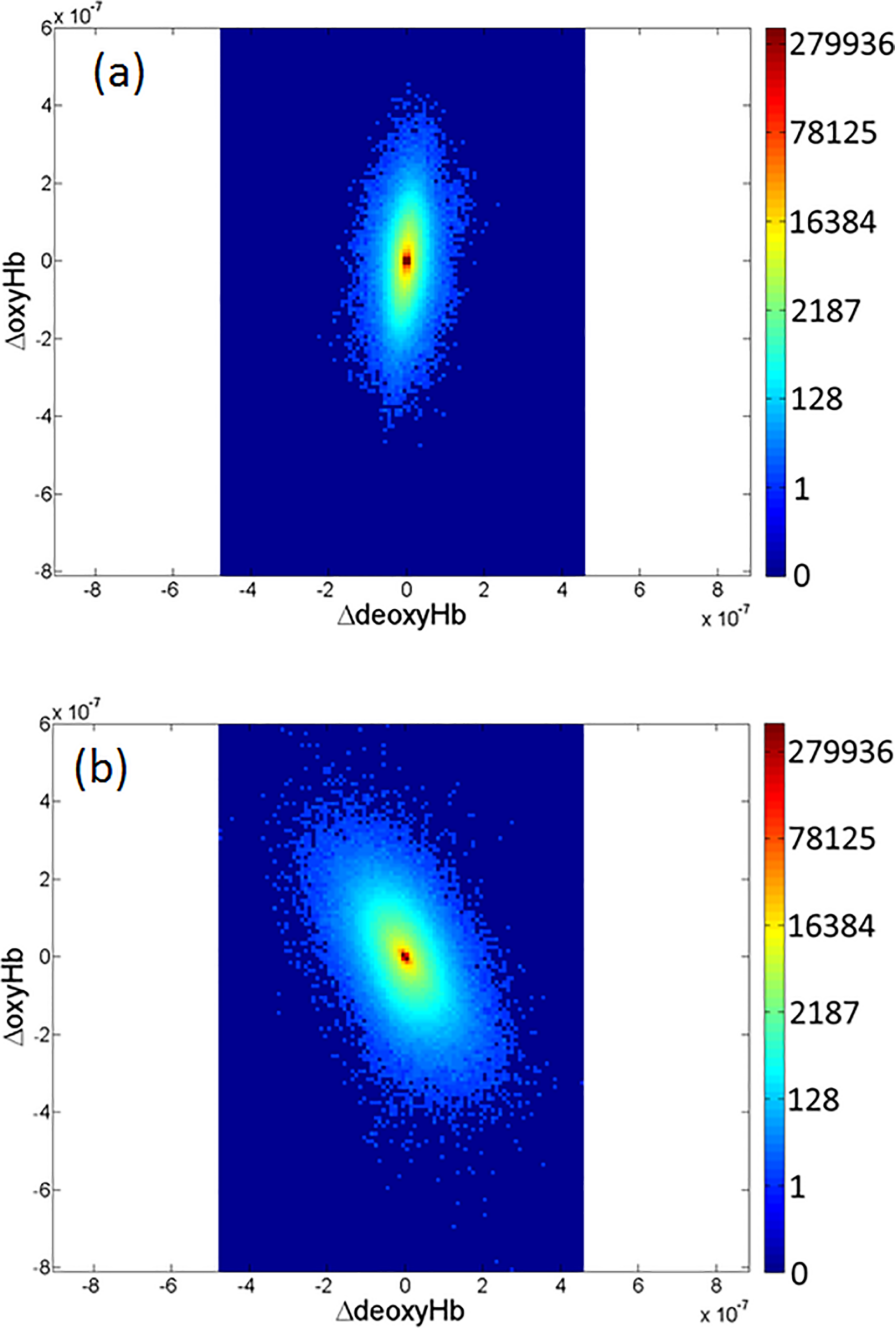
Resting-state ΔoxyHb and ΔdeoxyHb values for affected and unaffected breasts. Plots of superimposed whole-breast voxel trajectories of ΔoxyHb and ΔdeoxyHb for (a) unaffected and (b) contralateral cancerous breast from a representative clinical-study subject. [Subject was 50 y/o, BMI = 44, C size breast, with a 4 cm left-breast intraductal carcinoma].

Data shown in Fig 3 are representative plots of the voxel-dependent, time-varying Hb signal, displayed in the Fig 1 coordinate system, that includes contributions from the entire breast and measurement period. Seen are clouds of points, with the frequency of occurrence of each paired (ΔdeoxyHb, ΔoxyHb) combination indicated by the color scale. Inspection reveals distinct ellipsoidal distributions whose details strongly suggest sensitivity to the presence of disease.

While the overall patterns seen might suggest consideration of ways to quantify the shapes of the distributions, our focus is instead directed to the expectation that temporal variability in the actions of feedback mechanisms will serve to drive Hb-component values from one functional state to another. Accordingly, our attention is directed to quantifying features associated with such transitions.

### Hb state-transition coefficients

#### Coefficient maps of state transition rate values

**Fig 4:**
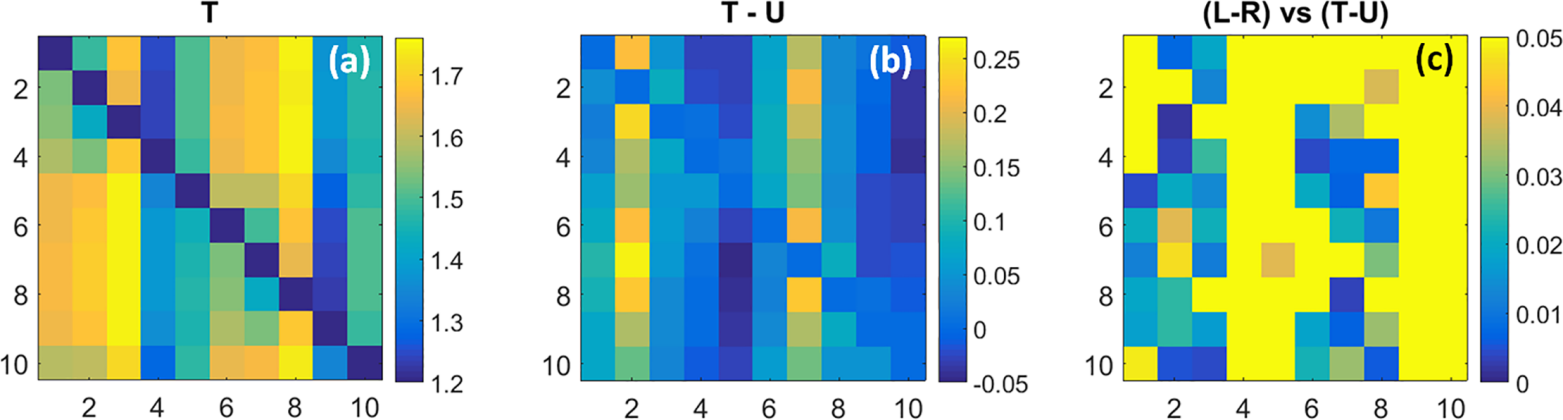
Coefficient maps of transition rate constants (*k*) and their comparison among subject groups. Plotted coefficients are computed by multiplying the Eq (3) result by the 1.8-Hz data sampling rate. (a): Transition rate constants (units are s^-1^) for the affected breast of subjects with unilateral breast cancer; (b): difference in coefficient values between tumor-bearing and unaffected contralateral breast; (c): p-values for (unpaired) t-tests comparing the inter-breast differences in coefficient values for subjects with and without breast cancer.

Fig 4 shows the group-mean values of the rate constants for inter-state transitions [Eq (3), multiplied by the data sampling rate] for women with breast cancer. Recall that each square cell’s column assignment identifies the state that is transitioned from while the row assignment denotes the state that is transitioned to. Fig 4(A) shows the rate-constant values obtained from tumor-bearing breasts (T), 4(b) the difference in values between affected and unaffected (U) breasts (T–U) and 4(c) p-values obtained from Student t-tests comparing the 4(b) data to the corresponding inter-breast differences [left-minus-right (L–R)] for the subjects without breast cancer (*i*.*e*., unpaired tests comparing L–R vs. T–U). As indicated by the color contrast, Fig 4(A) shows that transition rate constants for affected breasts vary over a range of ~1.20–1.75 second^-1^, depending on the transition pair. Closer examination reveals that the rate constants are preferentially elevated for transitions originating from States 3 and 8, and preferentially suppressed for those originating from States 4 and 9. Also seen is the apparent absence of an equivalent bias involving transitions into a given state (*i*.*e*., row features), indicating that rate constants involving reciprocal pairs of transitions are asymmetric.

A more careful inspection of Fig 4(A) reveals a distinct influence of the presence or absence of hyperemia on transition rate constants. The largest values are those for transitions from State 3 into States 5–9, and from State 8 into States 10 and 1–4. Common to these state transitions is a change in the algebraic sign of ΔtotalHb, suggesting sensitivity to hyperemia. Different from this are transitions from States 4 and 9. Here we find that those which do not involve an associated hyperemia (*i*.*e*., from State 4 into States 10 and 1–4, and from State 9 into States 5–8), have the smallest rate constant values overall.

Shown in Fig 4(B) are the differences between the group-level rate constant values for affected and unaffected breasts. Inspection reveals coefficient values that are preferentially elevated by the presence of disease and others that are reduced. Among the former, and most evident, are all transitions from States 2 and 7 into the others. Transition rates that are reduced in affected breasts principally include those originating from States 5 and 10. In Fig 4(C), we see that group differences are mainly clustered among transitions from States 1–3 and from States 6–8. Not explicitly revealed in Fig 4(C) is the additional finding that, for every transition type showing a significant inter-group difference, the inter-breast difference for the breast-cancer group is greater than that for the non-cancer group.

Taken together, these findings reveal two principal disease-dependent contrast features. First is the finding of elevated overall transition rates in affected breasts. We interpret this as consistent with the general observation that metabolic rates in tumors are elevated. Second, this behavior appears preferentially biased towards specific classes of transitions, in a manner suggestive of a compensatory response. In support of the latter is the finding that while transitions from States 3 and 8 have the largest values for either T or U breasts, the impact of disease appears greatest on either side of these transitions (*i*.*e*., largest positive and negative bias for transitions from States 2 and 7 and from 5 and 10, respectively). We also recognize that because measures were obtained under substantially steady-state conditions, a bias in one direction requires an opposing bias to not disturb the temporal mean value. However, given the many degrees of freedom associated with a ten-state model, it would seem evident that there are multiple ways that such a balance could be achieved, suggesting that a ‘salt-and-pepper’ pattern might be preferred in the absence of specific driving factors. The finding of distinctive patterns suggests that disease-dependent influences are present.

The transition-rate patterns for the L, R and U breast groupings are not explicitly shown, owing to space limitations and to their qualitative similarity to Fig 4(A). As an alternative, in Table 2 we have computed two quantitative indices of dissimilarity and have applied these to highlight group-level differences among the overall amplitudes and patterns of the measured coefficients. The first is a normalized root mean squared difference (nRMSD; a value of 0 indicates exact equality) between the coefficient amplitudes in the transition rate-constant maps (excluding the main diagonal), comparing either T and U or various pairings of unaffected breasts (*i*.*e*., L and R, L and U, R and U). The second is a measure of the spatial correlation of patterns observed between the rate-constant maps for the same pairings. To obtain a quantitative measure of the overall impact of breast cancer on rate constants, we have compared the mean value of the nRMSD and correlation for the three control group pairings (*i*.*e*., L and R, L and U, R and U) to the corresponding indices for T–U, in both cases using the t-score as a convenient indicator of group difference. This is intended to aid interpretation by presenting the contrast between tumor-unaffected and unaffected-unaffected values in terms of a familiar dimensionless unit, and not for the purpose of statistical testing.

**Table 2:** Analysis of group-level transition-parameter values for different subject-breast pairings.

Dissimilarity Index	Transition Parameter	x: T,U	y: mean(L,R; L,U; R,U)	z: SD(L,R; L,U; R,U)	(x–y)/z^(a^
nRMSD	*k*	0.063	0.029	0.012	2.81
	*P*	0.630	0.264	0.123	2.96
	*ϕ*	0.479–0.710	0.187–0.316	0.098–0.148	2.61–2.96
	*m*	0.778–0.924	0.343–0.438	0.166–0.210	2.28–2.62
Correlation	*k*	0.853	0.965	0.026	-4.27
	*P*	0.897	0.978	0.018	-4.46
	*ϕ*	0.988–0.996	0.997–0.999	0.00086–0.0028	-2.78 –-5.81
	*m*	0.952–0.975	0.983–0.993	0.0050–0.012	-2.61 –-4.29

Exact equality would produce nRMSD = 0 and correlation = 1. nRMSD is defined as: $nRMSD\left(\alpha ,\beta \right)=\sqrt{\sum {\left(\alpha -\beta \right)}^{2}}/\left[\left(\sqrt{\sum \alpha^{2}}+\sqrt{\sum \beta^{2}}\right)/2\right]$, where ‘*α*’ and ‘*β*’ are the sets of 90 coefficient values in the (main-diagonal-excluded) 10×10 maps for two selected subject-breast groups.

a) This dimensionless index is the t-score of the nRMSD or correlation value for the T,U pairing, in comparison to the mean and standard deviation of all the pairings of non-tumor breasts.

The correlation values obtained for measures of *k* are *r* = 0.853 for the T–U comparison, and *r* = 0.965 for the mean of the control pairings (SD = 0.026). While the former is notably reduced compared to the latter, indicating disease sensitivity, the amplitude patterns of transition rates over all state pairings among the breasts in either subject group is, nevertheless, strongly correlated. This is not overly surprising as in these instances the tabulated values are derived from data that was concurrently acquired from both breasts of the same subjects. Consequently, it is noteworthy that the L–U and R–U comparisons, involving data for two separate groups of subjects, also yield higher correlations than does the T–U case. The same pattern of results was found in the nRMSD values, which is larger (*i*.*e*., farther from zero, indicating less similarity) for the T–U case than for any of the control pairings, whether these involved comparisons of data from the same subjects (L–R) or from two distinct subject groups (L–U and R–U). The t-score values for nRMSD and correlation are 2.81 and -4.27, respectively, indicating that the impact of breast tumors is several times larger than the other sources of variance.

#### Coefficient maps of transition probability constants (*P*)

**Fig 5:**
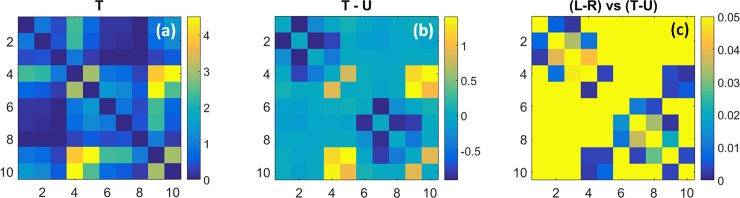
Coefficient maps of transition probabilities (*P*) and their comparison among subject groups. Plotted transition probabilities (units are percent) are computed using Eq (1). (a): data for tumor-bearing breast; (b): difference between tumor-bearing and contralateral, unaffected breast; (c): p-values for (unpaired) t-tests comparing the inter-breast differences for subjects with and without breast cancer.

Shown in Fig 5 are findings having the same format as in Fig 4, but for measures of State transition probability [Eq (1)]. A brief comparison of these to the corresponding parameter maps in Fig 4 reveals that the transition-probability and transition rate-constant patterns seen are weakly correlated and hence substantially independent. This finding is not surprising, as the prevalence and rate of change of behavior are independent in many systems. While many feature particulars are present, here we limit our consideration to only a few.

Most striking is the substantial symmetry among reciprocal pairs of transitions, over a larger range of values than is observed for the transition rate constants. Regions having the lowest probabilities correspond to transitions to and from States 1–3 and 6–8. Also seen is evidence of biases in the probabilities of transitions between states. For example, transitions either to or from States 3 and 8 are uniformly less probable than those involving adjacent states on either side (2 and 4, 7 and 9), producing a striped appearance. Having significantly higher, but also less uniform, probabilities are transitions to or from States 4, 5 and 9, 10. The net result of these biases is to produce a transition probability map that bears some similarity to the pattern of algebraic signs for transition-associated changes in ΔdeoxyHb concentration depicted in Fig 2. More specifically, it is seen that those transitions having the largest and smallest amplitudes mainly coincide with the regions that undergo a change in the algebraic sign of ΔdeoxyHb (yellow and navy-blue regions of Fig 2).

Similar to the difference between transition rate constants for affected and unaffected breasts [Fig 4(B)], results in Fig 5(B) shows that the probabilities of particular transitions can be either elevated or reduced in the T breast in comparison to U. One interesting difference between the rate and probability T–U maps is that the pattern of the latter appears clustered about three groups of transitions. Having reduced amplitudes are transitions involving State 2 with either 1 or 3 and those involving State 7 with either 6 or 8, while those having elevated amplitudes involve transitions between either State 4 or 5 and either 9 or 10, as well as directly between 4 and 5 or 9 and 10.

Included in Table 2 are values for the two inter-group similarity metrics described in the preceding section, computed for the transition-probability maps. The overall trends in these findings are similar to those for the rate constant measures. That breast cancer has only a modest effect on these indices for either *k* or *P* is not surprising, given that the average partial volume of tumor in affected breasts is on the order of 3% or less. Even so, the T–U nRMSD is appreciably larger than the mean value for the unaffected-breast pairings (t = 2.96 (*P*) and 2.81 (*k*)), and the correlation is substantially lower (t = -4.46 (*P*) and -4.27 (*k*)) for both types of coefficients.

While these findings indicate that the presence of disease does not grossly distort the mentioned coefficient values, evidence obtained from group difference measures for the T–U pairings [Fig 4(B) and Fig 5(B)] does reveal distinct biases in coefficient values among selected transition types. Notable here is the evidence that the effect of disease on the amplitude of *k* is substantially independent of its impact on *P*.

Fig 5(C) identifies those transitions for which the inter-breast probability difference is significantly different (p < 0.05) between the subject groups, using the same criterion applied in Fig 4(C). Inspection reveals that features that are significant mainly are those that have the largest group-amplitude differences. As in the rate-constant case, for every significant inter-group difference in Fig 5(C), the inter-breast difference for the breast-cancer group is larger than that for the non-cancer group.

#### Hb-state mean amplitude values

**Fig 6:**
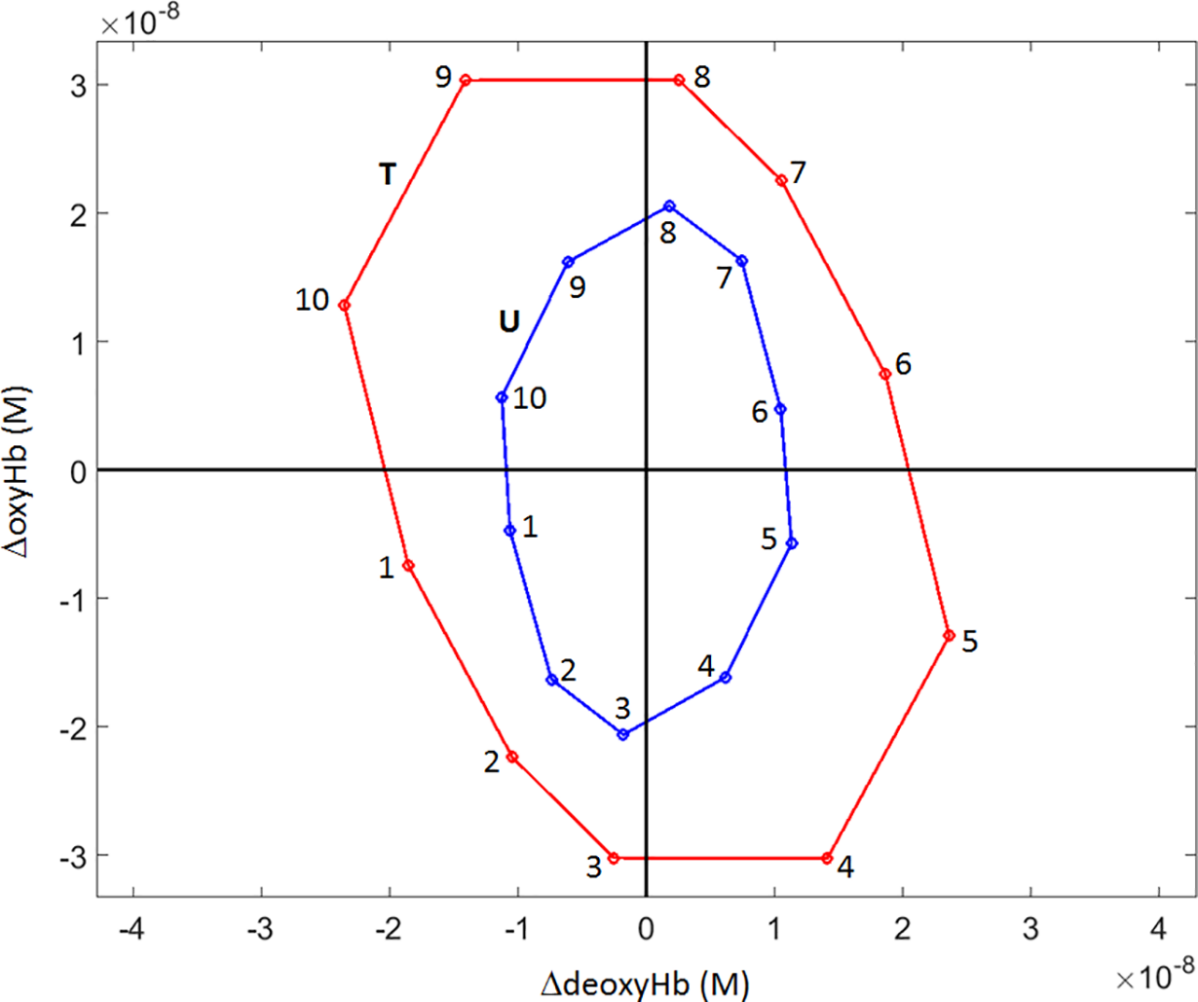
Hb-state dependence of mean values of ΔoxyHb, ΔdeoxyHb concentration. Plotted data points are average values across all time frames in each subject’s volumetric image time series and across all subjects in the breast-cancer group. T = tumor-bearing breast; U = contralateral, unaffected breast.

Additional state-dependent quantities that can be accessed via the applied methodology are measures of the mean Hb-component values for a given state, for the different subject groups. An example of these finding is shown in Fig 6, where the group-mean ΔoxyHb and ΔdeoxyHb values are plotted, for the affected (red) and unaffected (blue) breasts of affected women. Most evident is the shape of the distributions (mainly elliptical) and the obvious difference in their amplitudes. That the latter is greater for affected breasts is consistent with general finding of enhanced angiogenesis in tumors [15]. Also, as noted, the elongated trend in the direction close to that of the HbO_2_Exc axis for affected breasts indicates a disease bias that reflects the known degraded ability of the vasculature of tumors to maintain supply-demand balance by modulating blood volume to match changes in tissue oxygen consumption [31]. This trend is also consistent with the general finding of a greater degree of desaturation of Hb in tumors [31].

**Fig 7:**
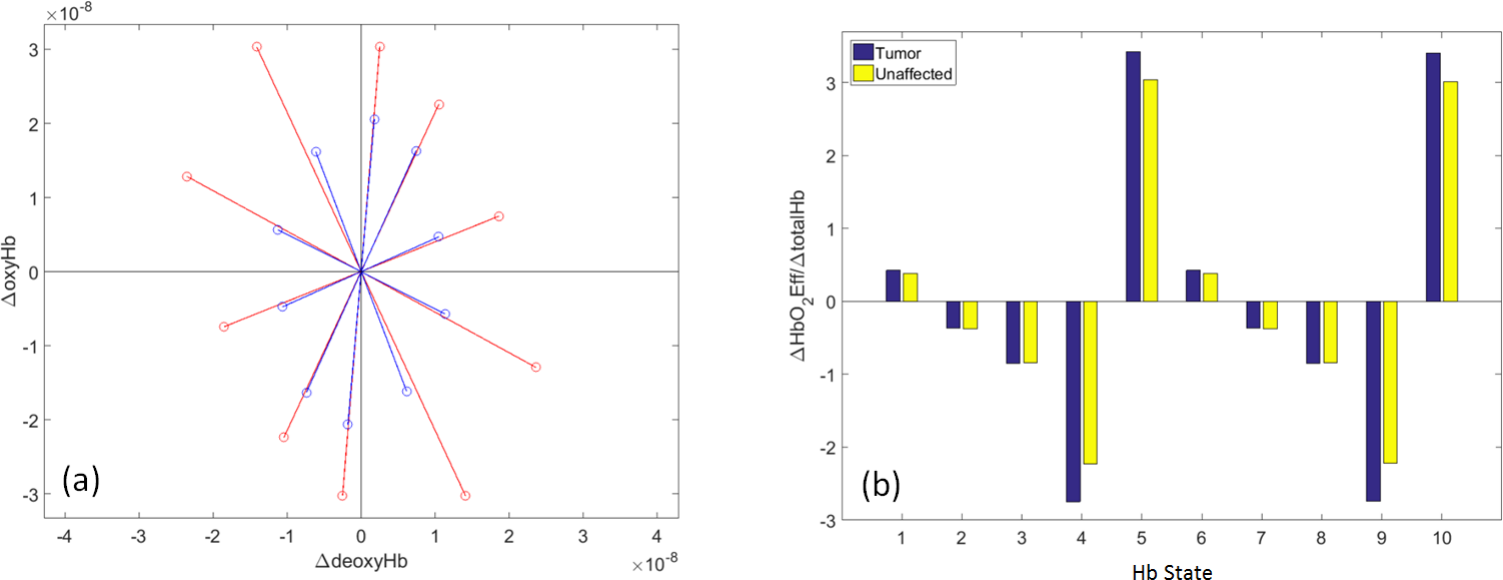
Replots of group-mean ΔoxyHb vs. Δ**deoxyHb data (Fig 6) for subjects with breast cancer.** (a) Each (ΔdeoxyHb,ΔoxyHb) data point (units are moles-L^-1^) is joined to the origin by a line segment, to emphasize the angular discrepancies between T (red) and U (blue) values. (b) Plot of the ratio (dimensionless) of each 7(a) data point’s projections onto the ΔHbO_2_Exc and ΔtotalHb axes (see Fig 1), showing the tendency of the T breast to mount a relatively smaller blood-volume response to changes in tissue oxygen demand.

A more quantitative examination of the phenomena revealed by the plot is presented in Fig 7. Here we begin [Fig 7(A)] by replotting the Fig 6 data points but, instead of connecting adjacent points with line segments to emphasize the elliptical patterns, we add line segments connecting each data point to the origin of the coordinate system. In this way, a property not obvious by inspection of Fig 6 is revealed: the angles between the red and blue line segments are close to zero for States 2, 3, 7 and 8, larger for States 1, 5, 6 and 10, and largest of all for States 4 and 9.

To evaluate this trend, we have investigated the values of the plotted points’ other Hb-signal components (*i*.*e*., their projections onto the other axes shown in Fig 1). The most informative result, plotted in Fig 7(B), is obtained by computing the ratio of ΔHbO_2_Exc to ΔtotalHb for each of the states (as previously noted, these are the components that are the most directly affected by the presence of a tumor: modulation of ΔtotalHb occurs to a proportionally greater extent in the unaffected breast, and of ΔHbO_2_Exc in the affected one). It is seen that for every state that has an angle discrepancy noticeable by inspection in Fig 7(A) (*i*.*e*., States 1, 4, 5, 6, 9 and 10), the absolute value of the ratio is larger for the tumor breast, which is to say that the magnitude of ΔtotalHb “response” relative to the magnitude of ΔHbO_2_Exc “stimulus” is smaller in the affected than in the unaffected breast [p < 1×10^−4^, for all listed states (paired t-tests)]. This finding is consistent with the known tendency of tumors to have a reduced capacity to regulate their blood supply to match changes in ambient oxygen demand [15]. We find it noteworthy that this effect is largest for States 4 and 9, which most closely represent the condition that supply is less(more) than adequate to meet demand, so that ΔoxyHb, ΔtotalHb and ΔHbO_2_Sat all have the same algebraic sign, and ΔdeoxyHb and ΔHbO_2_Exc have the opposite sign. Consequently, these states are the ones in which reduced capability of tumor-breast vasculature to match changes in blood supply to changes in oxygen demand would be most apparent, which is what we observe.

#### Hb-state partial volume values

**Fig 8:**
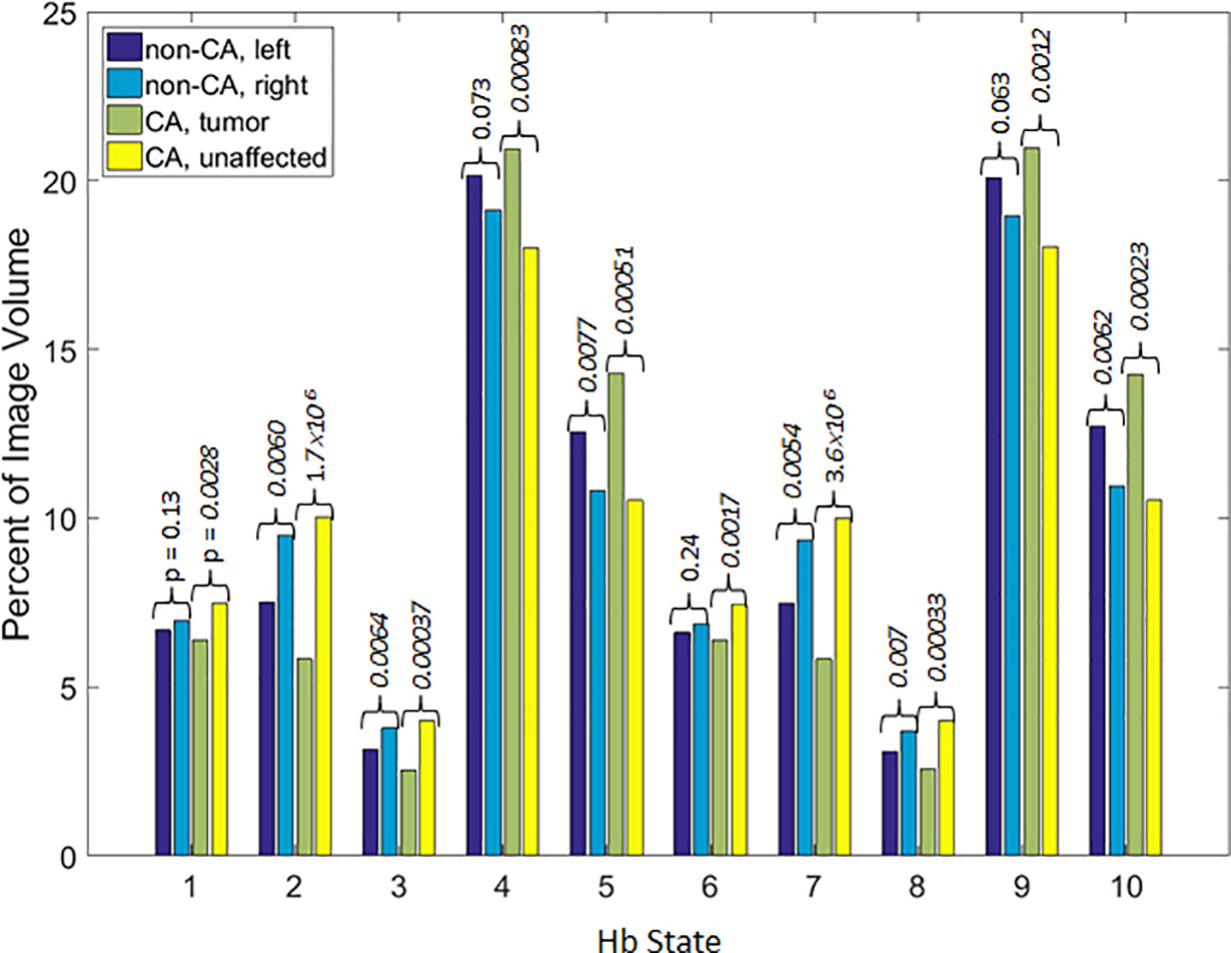
Percentage of image volume occupied by Hb states. Percentage of image volume in each Hb state, averaged across all time frames in each subject’s volumetric image time series and across all subjects in the indicated groups. Annotations are t-test p-values for comparisons of mean values for the left (L) and right (R) breasts (non-cancer subjects) and of the tumor (T) and unaffected (U) breasts (breast-cancer subjects).

A simple measure of state dependence is the time-average volume fraction of tissue that is occupied by a given state. This can be determined by counting the number of image voxels that reside in each state and expressing these quantities as the relative fraction (percentage) of the total volume, averaged over time. Fig 8 shows the ten states’ volume fractions (in units of percent) for the different groupings of study subject and breast-cancer status. Here we have separately plotted group-mean values for the L and R breasts of control subjects (*N* = 45), and for the T and U breasts of affected subjects (*N* = 18). Inspection reveals that the volume fraction for States 1–3 and 6–8 are reduced in T, while States 4, 5 and 9, 10 have increased values. We also note that while there is evidence of an intrinsic left-right bias in unaffected subjects, the p-values associated with this trend are consistently higher (*i*.*e*., lower statistical significance) than the findings obtained from corresponding inter-breast comparison for the affected subjects, even though the latter group contains less than half the number of subjects. We also note the diverse composition of the control group, which includes women who have no history of any breast pathology (*N* = 22) and a similar number of aged-matched subjects who have a variety of documented breast pathologies other than overt malignancy. This indicates that the observed bias exhibits disease specificity.

Separate from appreciation of the feature space of the volume fraction measures is a more quantitative understanding of relationships between these measures and the previously described State transition parameters (*i*.*e*., transition rates and probability). For instance, comparing the Fig 8 volume-fraction distribution to the pattern of transition probabilities in Fig 5 reveals that trends in the former appear to determine some of the features of the latter; *e*.*g*., that the lowest probability is found for transitions from/to States 3 and 8, and the largest is seen for transitions from/to States 4 and 9. However, there also are features of Fig 5 that would not be easily predicted from inspection of Fig 8, such as the greater probability for transitions between 4 and 10 or between 5 and 9 than for ones directly between 4 and 9. It is worth considering what may be the form of the function that transforms the 2D probability map into the 1D volume fractions, and how consistent it is across the four subject-breast pairings.

The simplest assumption is direct proportionality between transition probability and volume fraction, with the rate constants serving as the constants of proportionality. Thus, the volume fraction for State *j* would be equal to

$$V_{j}=K\sum_{i=1}^{10}\frac{P_{ij}}{k_{ij}},\;K\equiv \frac{100}{\sum_{j=1}^{10}\sum_{i=1}^{10}\frac{P_{ij}}{k_{ij}}},$$ (12)

where the normalization constant *K* ensures that the quantity on the right-hand side of Eq (12) is equal to 100 when summed over all *j* (as is the sum of the *V*_*j*_s, by definition). It is found (not shown) that there is excellent agreement between the true and computed volume fractions (*i*.*e*., left- and right-hand sides of Eq (12), with a mean relative percent discrepancy (*i*.*e*., $100\left(V_{j}-K\sum_{i=1}^{10}\frac{P_{ij}}{k_{ij}}\right)/V_{j}$), of just over 1% across the four subject-breast groups. This finding of consistency across data sets demonstrates the stability of the data transformations used to derive the state-transition coefficients.

In addition, the observed accuracy of the Eq (12) model shows that transitions between Hb states are first-order processes, as kinetics of any other order would lead to a different mathematical relationship among *k*, *P* and *V*. In view of the mathematical relationship between rate constant and mean lifetime [Eq (3)], the 1/*k*_*ij*_ factor in Eq (12) can be replaced by *τ*_*ij*_, which leads to a compact formula relating the volume fractions of all states to the transition-probability and mean-lifetime coefficients:

$$V=K\cdot diag\left(P^{T}\tau \right),$$ (13)

where *K* is the same as in Eq (12), *diag*(**X**) is a vector consisting of the main-diagonal elements of the matrix **X**, and *P*_*ij*_ and *τ*_*ij*_ are the *ij*^th^ elements of **P** and *τ*, respectively.

The preceding relationships noted, the added dimensions of the transition-rate and probability spaces relative to the volume fraction nevertheless provides an opportunity to appreciate other disease-dependent biases. Of interest is the noted substantial independence of disease impact on specific features of these transition spaces (*e*.*g*., different particular transitions are most strongly affected in the *k* and *P* results). We contrast this finding with our previous report that explored the same imaging data and demonstrated a prominent temporal feature from affected breasts that is consistent with the existence of a persistent inflammatory response in tumors in response to upregulation of NO formation [16]. While the data treatment strategy pursued here does not support independent assessment of behaviors originating from the tissue and vascular spaces, it is difficult to reconcile the evidence of differential influences on the State spaces on the basis of only known effects of NO on the vascular space. Consequently, we interpret this behavior as consistent with the actions that NO is known to have on the tissue space as well (*e*.*g*., NO is a known competitive inhibitor of oxygen binding to cytochrome c-oxidase [32]), combined with expected compensatory effects.

To briefly summarize, in this section we have identified the principal parameters accessible from measures of the Hb State-transitions. Comprising 2D spaces are the transition rate and probability coefficients, whose details appear mainly independent of each other and also appear disease-sensitive in a manner consistent with the several prominent cancer phenotypes. An additional finding is evidence of good feature recovery (*i*.*e*., computed volume fraction) from first-order behavior in the kinetics of the rate-weighted transition probabilities.

In the next section we present findings from measures of the Hb-component transition flux, which represents an even more feature-rich information space.

### Hb-component transition coefficients

#### Coefficient maps of Hb-component flux amplitudes

**Fig 9:**
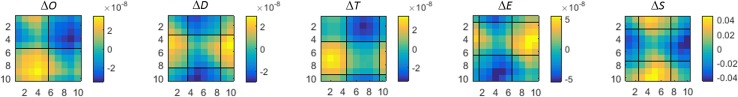
Intrinsic transition flux for subjects in the non-breast-cancer group. Intrinsic transition flux [i.e., average change, per transition, in concentration (units are moles-L^-1^) or saturation (units are %); computed using Eq (6)] results for the control-group subjects. Graphed data are averages over the left and right breasts. Headings denote the Hb components as indicated in Table 1. Thin solid lines separate regions according to the algebraic signs of the pre-and post-transition Hb-component values (see Fig 2).

Shown in Fig 9 are the state-dependent amplitudes of intrinsic transition flux [Eq (6)] for the different components of the hemoglobin signal, computed from image time series of the breasts of control subjects (*N* = 45). These quantities, which can have either positive or negative values (*i*.*e*., increased or decreased flux), correspond to the average difference between the transition-dependent component values in the initial time frames of pre- and post-transition time intervals. Note that the scale for ΔHbO_2_Sat is expressed as change in percent, while the values for the other components are molar quantities. Also, recall that these quantities are computed on a per-transition basis and not over a defined time interval, and that the plotted quantities are obtained by averaging over the full volume of the breast, the 5–10 minute period of observation and for all control-group subjects. Inspection of the figure reveals several prominent features.

One feature clearly present is the tendency of the amplitude changes to appear spatially correlated with the component-dependent algebraic sign patterns that accompany given transition types (see Fig 2). This is especially evident for the ΔtotalHb component. While application of a simple differencing scheme can be expected to produce a pattern whose deviation from the temporal mean is sensitive to the pattern of algebraic-sign changes corresponding to the transitions, this is not sufficient to guarantee a smoothly varying pattern for any given component.

The counter consideration is one where each amplitude within a specified sector [*e*.*g*., all transitions in which the algebraic sign of the component amplitude change from positive to negative (*i*.*e*., dark blue regions in Fig 2)], would be mainly independent of those of neighboring transition types, thus producing a “salt-and-pepper” appearance. This outcome would have important consequences for our postulated relationships between a sequence of state transitions and their sensitivity to intrinsic biological factors. As noted in Methods, Section 5, the numbering sequence we have assigned to the Hb states reflects a belief that passing through them in order traces out the compensatory hyperemic response to a hypothetical supply-demand imbalance. Thus, transitions between adjacent states (*e*.*g*., transition 2→3) are expected to reflect a lower degree of imbalance than are transitions involving reciprocals (*e*.*g*., transition 1→6). It follows that if the transition-flux pattern were to have more of the conjectured salt-and-pepper appearance, it would suggest that either the assigned sequence of state transitions does not reflect the hypothesized interpretation, or that our findings are strongly biased by unanticipated driving factors. Conversely, we interpret the smoothly varying patterns that were obtained as evidence that the assigned state transition sequence does roughly coincide with expected gradients in supply-demand imbalance. We further note that this understanding also holds should measures of flux be alternatively expressed on fixed-time basis (sec^-1^), which is a finding that expectedly follows from appreciation that the amplitude range for transition rates (Fig 4) is many-fold smaller than that seen for intrinsic flux (Fig 9).

For completeness we provide a quantitative summary of comparisons between the intrinsic-flux measures for different pairings of breast and subject group, similar to those already considered for the rate constant and transition probability findings. Data in flux-related nRMSD and correlation rows of Table 2 indicate that while gross trends across all parameter transitions are highly correlated among the different groups, the range of intrinsic-flux correlations for Hb components is notably lower for the T–U pairing than for any of the others, and the nRMSD is notably larger.

**Fig 10:**
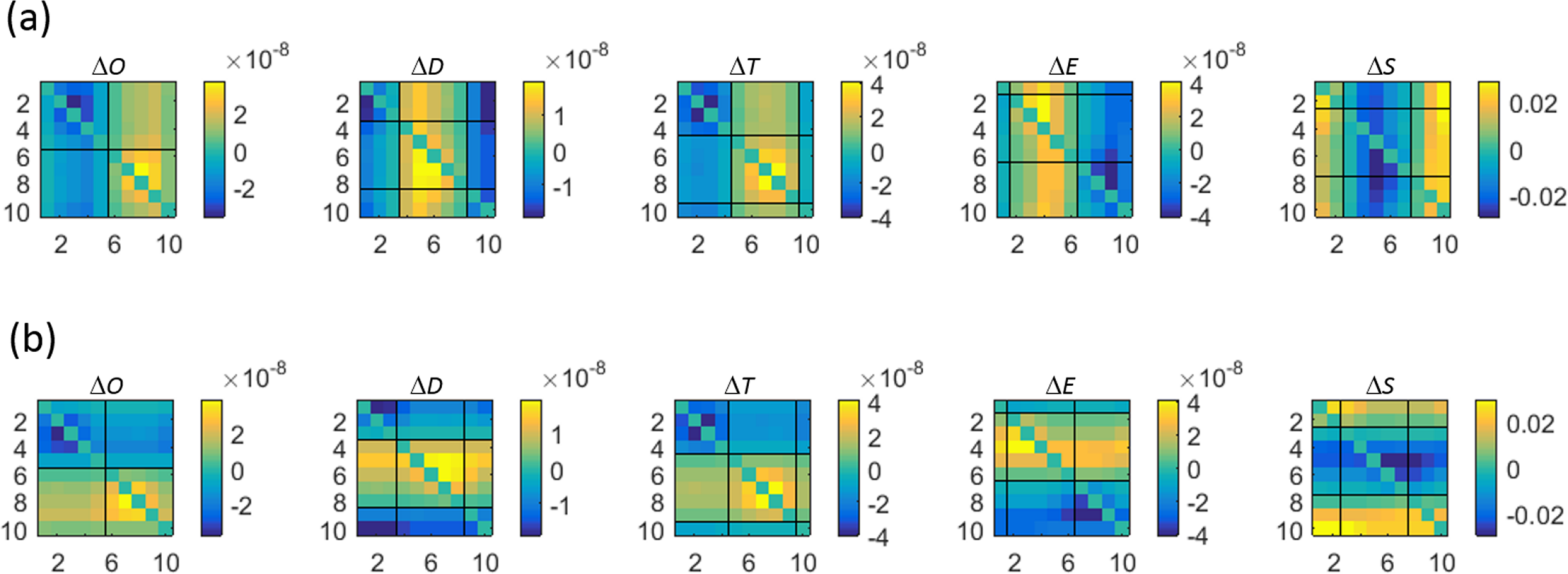
Average ΔHb amplitudes in the pre- and post-transition image time frames for data in Fig 9. (a) Average ΔHb amplitude in the pre-transition state encoded by the column index, given that the transition that ultimately occurs is to the State encoded by the row index. (b) Average ΔHb amplitude in the post-transition state encoded by the row index, given that the transition that ultimately occurs is from the State encoded by the column index. Headings denote the Hb components as indicated in Table 1; units are percent for ‘s’ and moles-L^-1^ for all others. Thin solid lines separate regions according to the algebraic signs of the pre-and post-transition Hb-component values (see Fig 2).

Returning to Fig 9, a more granular examination of the contrast features reveals that while amplitudes do smoothly vary, there also is evidence that different components of the hemoglobin signal exhibit preferred transition types. To gain a better understanding as to the factors that might serve to facilitate these preferences, we have made separate examinations of the amplitudes of each component during the pre-transition and post-transition time frames, for measures obtained from control subjects. These results are shown in Fig 10.

Plotted in Fig 10(A) is the average amplitude, relative to the temporal mean, of the concentration or saturation at the beginning of the pre-transition time interval, while Fig 10(B) shows the corresponding values at the start of the post-transition interval. Inspection reveals two prominent features. First, the pattern of amplitudes differs for the different components. Also evident is that, while the corresponding pre–and post–transition state amplitude maps appear mainly as conjugate pairs (*i*.*e*., each is nearly the transpose of the other), there is a notable relationship between regions in the coefficient maps having the greatest deviation from the mean, and the associated flux amplitude identified in Fig 9. In particular, we observe that large-amplitude features in the former category are, in every instance, roughly spatially correlated with regions in the latter that have reduced flux amplitudes.

The observed relationship between fluxes and the pre- and post-transition amplitudes invites a comparison to a body of well-established theory on factors influencing thermodynamics and kinetics of chemical reactions, and, in particular, the influence of enzymatic transformations. For instance, reactions that have low potential energy barriers tend to occur more often than those with higher barriers. It follows that the patterns of high and low flux regions seen in Fig 9 exist because the pre–and post–transition amplitudes are proportional to “intrinsic energy barriers” that serve to produce these patterns. We present this interpretation in the context of an analogy, with the understanding that the true mechanism is likely complex and dependent on a host of factors that may or may not explicitly depend on the suggested mechanism. Continuing with the chemical reaction analogy, the relation considered here (between intrinsic fluxes and the pre- and post-transition concentrations) calls to mind yet another phenomenon relevant to chemical reactions, the property of mass action.

The Law of Mass Action states that the rate of a chemical reaction is directly proportional to the product of the concentrations (activities) of its reactants. Extension of this understanding to the current study suggests that the observed transition fluxes are in some way proportional to the amplitude patterns shown in Fig 10. This hypothesis can be evaluated by regressing the intrinsic transition flux against the corresponding change in average amplitude (*i*.*e*., concentration or saturation) between the pre-transition and post-transition states. These results are in shown in Table 3. Inspection shows that these quantities are strongly correlated, but differ in their slopes (ranges for the corresponding intercepts are 10^−6^–10^−5^ for ΔHbO_2_Sat and 10^−12^–10^−10^ for the others, and in no case is the intercept significantly different from zero).

**Table 3:** Linear regression parameters for fits of intrinsic flux to average difference between post- and pre-transition ΔHb levels.

Parameter	Intrinsic flux regressed on Average concentration (or saturation) difference							
		L	R	T	U	x: mean(L,R,U)	y: std(L,R,U)	(T–x)/y
**Correlation**	**Δ*E***	0.969	0.969	0.981	0.968	0.968	0.00065	19.5
	**Δ*D***	0.965	0.966	0.977	0.970	0.967	0.0028	3.5
	**Δ*T***	0.994	0.994	0.996	0.993	0.994	0.00070	3.9
	**Δ*S***	0.980	0.981	0.986	0.983	0.981	0.0016	3.02
	**Δ*O***	0.953	0.959	0.965	0.965	0.959	0.0063	1.03
**slope**	**Δ*E***	0.86	0.84	0.92	0.82	0.84	0.022	3.7
	**Δ*D***	0.85	0.83	0.90	0.83	0.84	0.011	5.3
	**Δ*T***	0.54	0.54	0.60	0.56	0.55	0.014	4.1
	**Δ*S***	0.93	0.91	0.95	0.91	0.91	0.010	3.9
	**Δ*O***	0.65	0.64	0.73	0.63	0.64	0.0086	10.9

Linear-regression slope and correlation-coefficient values obtained from fitting values for intrinsic flux to the average differences between the ΔHb levels in the post-transition and pre-transition states, for each Hb component.

The existence of a linear dependence implies that the fluxes are driven by first-order processes. Nevertheless, as noted, because the applied methodology does not distinguish among contributions from the principal tissue spaces to the observed behaviors, it seems likely that the identified order is an amalgam of more complex behaviors that actually do occur in these spaces. While this trend is seen in both subject groups, the finding of elevated slope values across all components of the hemoglobin signal for the affected breast of the cancer group indicates a disease bias. Further, the observation that the ΔHbO_2_Sat intrinsic flux is most sensitive to the gradient (*i*.*e*., slope is closest to unity) suggests that changes in blood oxygenation are favored over changes in blood volume to achieve steady-state supply-demand balance. This finding is also consistent with the qualitatively similar behavior associated with the ‘stimulus-response’ trend identified in Fig 7. Different here, however, is the added finding that driving this bias is a concentration dependence consistent with a first-order process.

#### Influence of disease on pre- and post-transition component amplitudes

**Fig 11:**
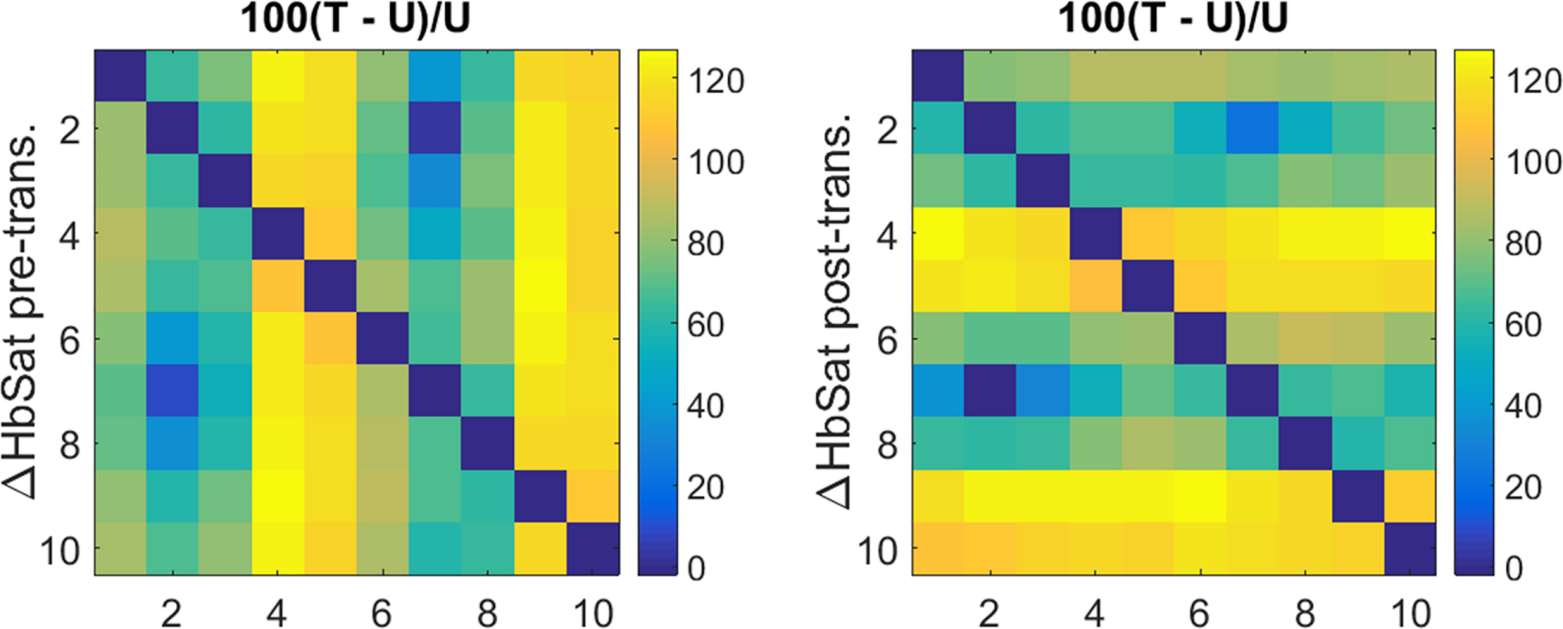
Impact of breast tumor on pre- and post-transition ΔHbO_2_Sat amplitudes. Relative percent difference (dimensionless) between the (left) pre-transition and (right) post-transition ΔHbO_2_Sat amplitudes, comparing the tumor-bearing and unaffected breasts of subjects in the breast-cancer group.

To gain additional understanding of the observed disease bias on what appears as a mass-action effect, we have computed the inter-breast relative differences for both the pre- and post-transition component amplitudes, and have compared these relative differences across subject groups. The full comparison generates two parameter maps (one for the pre- and one for the post-transition amplitudes) for each component and subject group, and for brevity we limit our presentation of findings to only the ΔHbO_2_Sat component. These results are shown in Fig 11. Inspection reveals several prominent findings.

The pattern of features in Fig 11 differs qualitatively from those seen in any of the other feature maps. At the grossest level of inspection, we find that each of the identified parameter spaces [(*i*.*e*., State transition rate constants (Fig 4) and probabilities (Fig 5), intrinsic flux (Fig 9), pre- and post-transition component amplitudes (Fig 10) and the associated sensitivities to disease (Fig 11)] appear mainly independent of each other, especially at the level of individual State-component transitions. This finding is striking and strongly suggests that the composite of intrinsic driving factors that serve to maintain steady-state supply-demand balance in tissue exhibits differential sensitivity to each of the elements of the hemoglobin signal. However, the fact that three of these components, namely ΔHbO_2_Sat, ΔtotalHb and ΔHbO_2_Exc, are not independent of the amplitudes of ΔdeoxyHb and ΔoxyHb, suggests a degree of fine tuning that, while grossly evident from the understanding that modulation of blood delivery to tissue is continuous, has nevertheless been difficult to quantify in the context of specifically observable parameters.

Returning to the details of the parameter maps in Fig 11, inspection reveals additional features that warrant comment. In the results for the breast-cancer subjects, we observe that transitions to and from States 4, 5 and 9, 10 are the ones exhibiting the largest inter-breast relative difference. This shows that the pre- and post-transition amplitudes of these quantities have the greatest sensitivity to the presence of disease. Having the lowest disease sensitivity are transitions between States 2 and 7. Not shown are grossly similar findings involving patterns seen for the other Hb components. Thus we find instances where the response to disease varies from a more general influence on a class of transitions to one where the absence of influence is mainly limited to a single transition type. Additionally, the observation that essentially all metric values listed in Fig 11 are positive indicates that, similar to the findings for transition rates (Fig 4) and consistent with the general phenomena of elevated metabolic rates in tumors, flux rates are also elevated in the tumor bearing-breast.

#### Hb-flux coefficient trends among Hb components

While the above findings serve to identify a relative independence of responses across the different feature spaces for individual Hb components or States, the high dimensionality makes it is difficult to appreciate specific trends among the various classes of information. Should such trends lend themselves to defining dependencies that are amenable to simplified measures, they could have the potential to serve as concise markers of disease. [A brief examination of this is considered in S5 Appendix.] Of note here is a demonstration that, while the previously suggested simplified measures of the data clouds in Fig 3 may have limited value, similar efforts applied to the granular representations of data spaces afforded by the applied finite-state methodology appear far more promising.

#### Amplitude maps of weighted transition mass values

**Fig 12:**
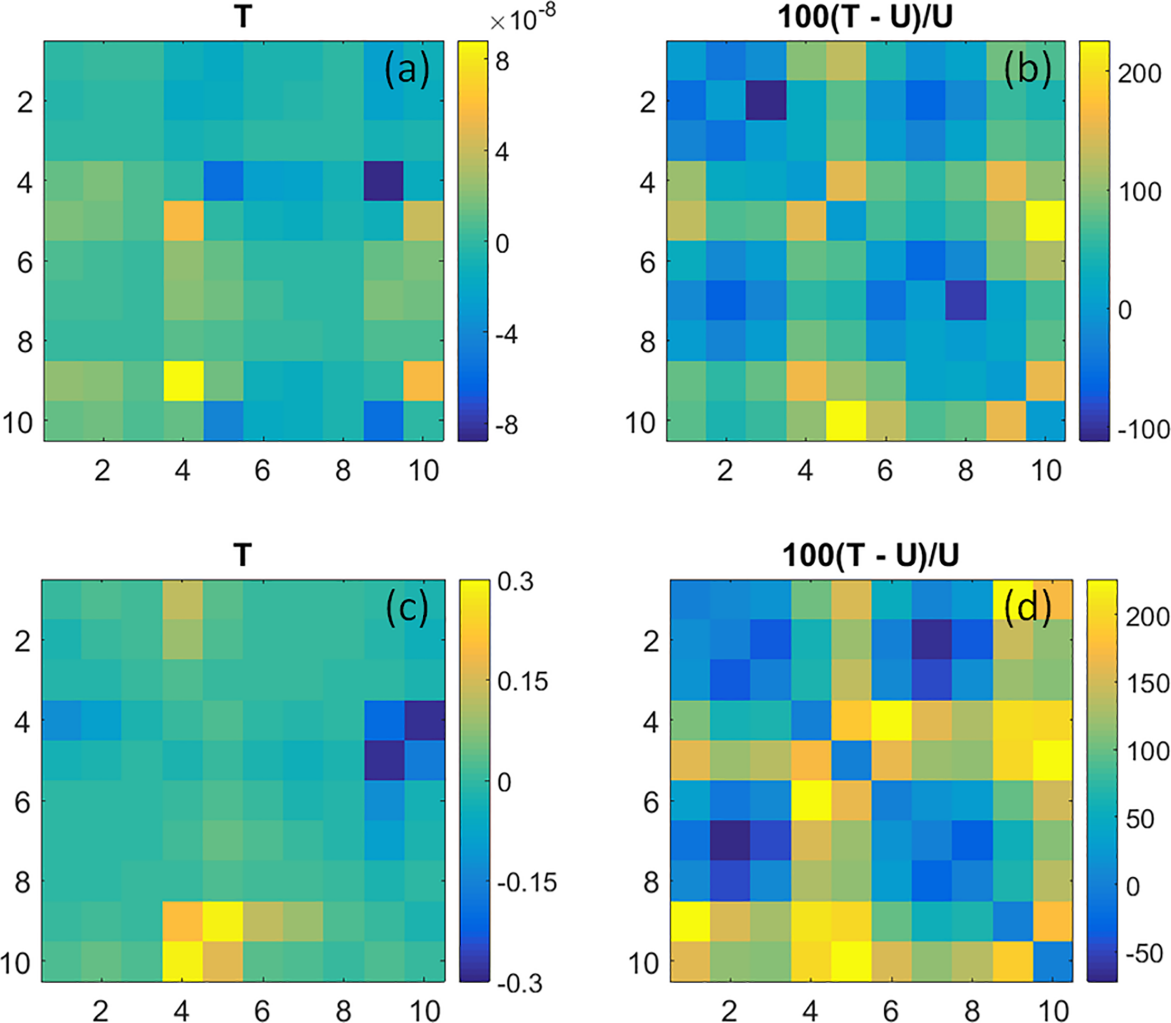
Transition-mass findings for the breast-cancer subject group. (a), (c): Transition mass of ΔtotalHb (moles-L^-1^) and ΔHbO_2_Sat (%), respectively, for the tumor-bearing breast of the breast-cancer subjects; computed using Eq (7). (b), (d): Relative percent difference (dimensionless) between the transition masses for ΔtotalHb and ΔHbO_2_Sat, respectively, comparing the affected and unaffected breasts of the same subjects. Principally distinguishing the transition responses seen is that the dominant transitions observed for ΔtotalHb (10→5, 4→9, 10→9, 4→5) involve an obligatory hyperemic response, while those for ΔHbO_2_Sat (4→10, 5→9) do not.

As noted in Methods, we recognize that composites of coefficient amplitude values [Eqs (7)–(9)] have the potential to reveal other features not readily appreciated by examining the intrinsic coefficients. One feature of interest is the quantity we refer to as the ‘transition mass’, which is the product of the intrinsic flux amplitude for a given component and the transition probability. This quantity holds significance, as it best describes the overall quantity of Hb component that participates in transitions of a particular type. Because the transition probability (Fig 5) varies significantly over the set of transition types and is weakly correlated with the patterns seen in the component flux maps (Fig 9), it can be expected that the corresponding weighted-flux coefficient map will also differ significantly from its unweighted counterpart. Shown in Fig 12 are selected plots of this coefficient space for ΔtotalHb and ΔHbO_2_Sat.

Plotted are 10x10 maps of the transition-mass amplitude for tumor-bearing breasts, and the relative difference in amplitude when this is compared to the corresponding data for the contralateral unaffected breast. Findings for ΔtotalHb and ΔHbO_2_Sat are shown in Fig 12(A), 12(B), 12(C) and 12(D) respectively. Inspection of these weighted flux maps reveals a transition dependence that differs substantially from those for the intrinsic-flux amplitudes (Fig 9, and Figure G in S5 Appendix). Here we find that only a selected few transition types appear to dominate the overall pattern. It is also apparent that the distribution of these among the various transition types differs between the two Hb components. Favored for ΔtotalHb [Fig 12(A)] are transitions mainly among States 4, 5, and 9 and 10. In contrast, the pattern seen for ΔHbO_2_Sat [Fig 12(C)] strongly favors transitions between either 4 or 5 with either 9 or 10, with reduced amplitudes for adjacent transition types. Patterns grossly similar to the latter are found for the other Hb components (not shown). Also shown in Fig 12 is the pattern of relative changes in transition mass when the affected and unaffected breast patterns are compared [12(b),(d)]. Inspection reveals that the dependence on transition type is qualitatively different from similar comparisons presented above (*cf*., Fig 11). Common to both Hb components are regions where the relative difference value is depressed in the affected individuals and others where it is notably increased. As measures of *m* constitute a composite quantity that includes *P*, it is not overly surprising to find that trends in bilateral breast comparisons of affected subjects are grossly similar to that seen for *P*. Nevertheless, the details of these trends differ among the different coefficient classes [*cf*., Fig 5(B)], indicating that the influence of disease varies across the coefficient spaces. Also, and similar to findings noted in Figs 6, 7 and 11, computation of the average relative differences in transition mass (Fig 12B and 12D) shows that these are elevated in the tumor breast, with a bias favoring ΔHbO_2_Sat compared to ΔHbtotal (99% and 48% increase, respectively).

Data in mass-related nRMSD and correlation rows of Table 2 indicate that, similar to the previously considered intrinsic fluxes, the transition-mass correlations for all Hb components are notably lower for the T–U pairing than for any of the others, and the nRMSD is notably larger.

### ROC findings

**Fig 13:**
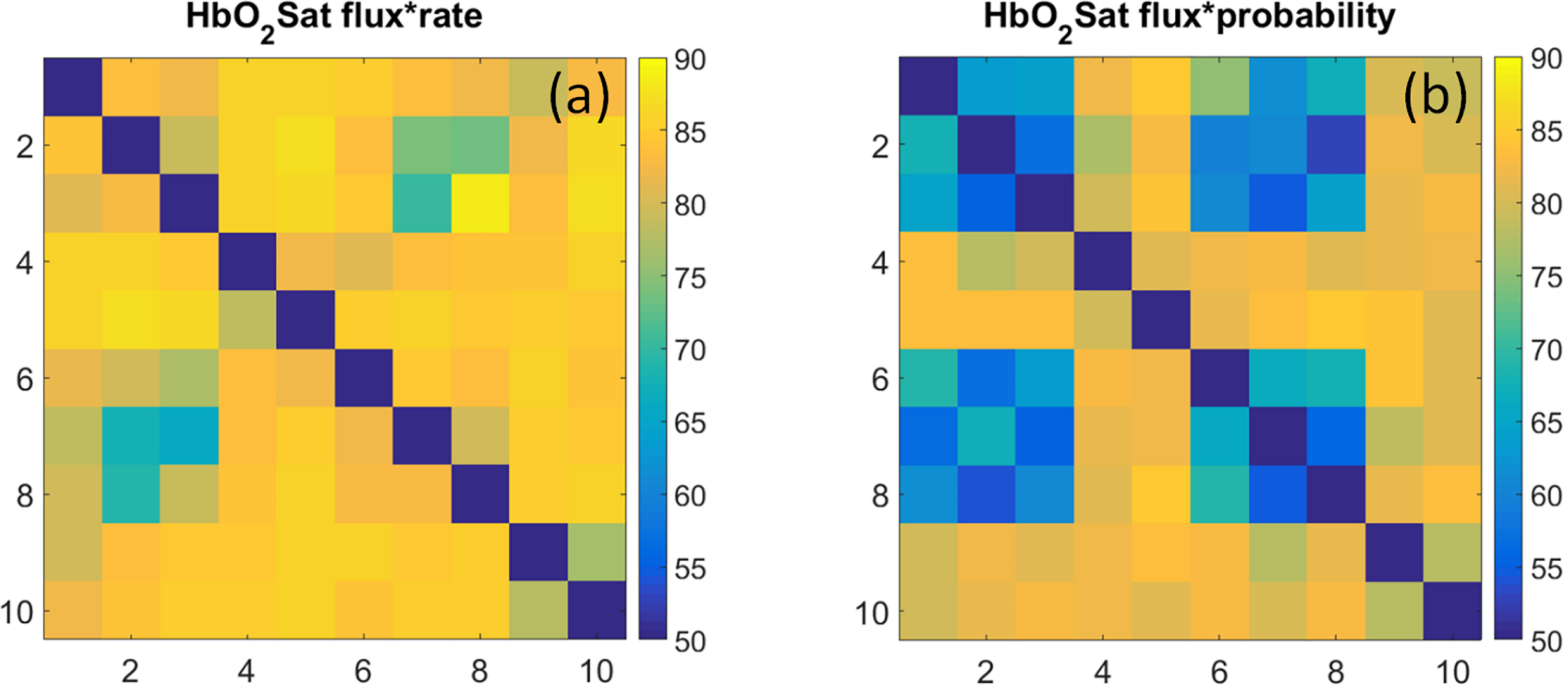
Exemplary ROC-analysis findings. Area-under-curve values from ROC analyses (units are percent) comparing the inter-breast differences for subjects in the non-cancer and breast-cancer groups. The transition-based coefficients used as input to the ROC analysis computations are the product of intrinsic flux and either transition rate constant (a) or transition probability (b).

Finally, shown in Fig 13 are results from ROC analyses (univariate AUC measures) applied to two classes of weighted coefficient values for ΔHbO_2_Sat: flux amplitude weighted by the rate constant [Eq (8), Fig 13(A)], and the transition mass [Eq (7), Fig 13(B)]. Inspection reveals that for the former most of transition coefficients exhibit AUC values in the 80–90% range. Different from this is a distinct checkerboard pattern seen in the transition-mass case. We consider this finding as additional evidence that the various elements of the coefficient space examined carry qualitatively different information.

## Discussion

A network, whether physical or conceptual, is composed of a collection of interacting components and serves as a vehicle of information transduction. The information content can be physical, ranging in size from electrons to molecules to macroscopic objects, or non-physical as in communication networks. Networks also may or may not have well-defined physical structures. The former category includes roads and telephone lines, and, in the case of biological systems, defined anatomical features such as the vascular tree or peripheral nervous system. There also are many examples in biology that are more similar to communication networks, in that coordinated activities occur without the need for specific reliance on defined pathways. Examples include actions of hormones and other effectors. Also, it is well appreciated that, on a systems level, biological organisms rely on network behaviors to maintain homeostasis. Often this takes the form of actions mediated by feedback mechanisms.

Consideration of two independent factors bridges these understandings of systems-level biology to our goal of enhanced detection of breast cancer with the NIRS technique. The first builds on a prior report from our group, which demonstrated that promising detection of breast cancer is achievable from measures of a particular feature whose origin has long been associated with tumor growth [16]. Evidence of enhanced modulation of intrinsic signals was identified, the spatial dependence of which appears to extend far beyond the tumor border, even in the case of small tumors. Also seen was evidence that the temporal features of this behavior were substantially in line with findings from a prior report obtained using dynamic thermography methods [33]. In that report, strong evidence was provided that the origin of the observed temporal behavior was attributable to the actions of enhanced NO production, a feature commonly associated with the sustained inflammatory phenotype of most tumors [34]. Thus, in addition to being the causative factor driving the observed temporal features, this property of NO, which is a highly diffusible gas, may also serve to explain the extended spatial features seen as well.

While the identified features are of interest in their own right, the recognition that they were observed under resting-state conditions has prompted us to revisit the more basic issue of how measures of this type can be explored to reveal useful findings. This leads to the second consideration that has motivated the current work.

A simple observation that we previously recognized [18] is the tendency of most reports involving NIRS-measures of tissue to explore the time-varying behaviors of the Hb components separately, and not as a co-varying system. Indeed, this approach was adopted even by us in our prior report exploring resting state behaviors in subjects with breast cancer [16]. To align this consideration with our current work, it is helpful to briefly digress and take a broader view of the analysis strategies applied to the exploration of behaviors of complex systems. This goal is pursed from two different vantage points.

As previously noted, data interpretation strategies fall into two principal categories, strong and weak models, each with advantages and tradeoffs. While the latter are often more flexible, the utility of a given approach can be situation-dependent. Thus we hold that our choice of adopting measures under the resting state is likely to provide for more repeatable outcomes than would more complex protocols. We also note that the principal phenomenology observable in the resting state follows from actions of feedback mechanisms. Significantly, experience from clinical medicine has shown that disturbances in these behaviors are often an early sign of disease [35].

Returning to the current work, having observed that disease features are present in the spatial and temporal domains and that components of the Hb signal do co-vary, we appreciated the merits that a classic weak-model approach based on a finite-state formalism can bring to exploring this composite of behaviors. A simple representation of this is available from examination of a FMC. Considered here are discrete states of a system that undergoes transitions from one state to another in accordance with some defined connectivity. Application of this formalism to our measures of the Hb signal is contingent upon satisfaction of two requirements. One is the need to include descriptions of co-varying behaviors within our state definition. The second is the need to reduce continuous measures to a simplified discrete representation.

The first condition was met by choosing a state definition that includes some set of behaviors that comprises all five components of the hemoglobin signal simultaneously. The second requirement was met from a simple appreciation that the experimental conditions involved recording in the resting state. Because intrinsic behaviors tend to oscillate about a temporal mean under these conditions, we appreciated that a first-order criterion for defining a state can be accomplished by reducing the continuously varying amplitude of each Hb component to a binary variable: alternately above and below the temporal mean value. This leads to an assignment of ten distinct states, each corresponding to a unique permutation of algebraic signs of the five components.

While the above considerations lead to unique state assignments, still missing is a set of criteria that captures features associated with the transition matrix, which is the principal phenomenology that finite-state formalisms are intended to explore. Motivating our approach was the understanding that feedback mechanisms likely operate with different time constants in a spatially and temporally varying manner. Having the aim of retaining simplicity in this first application, we appreciated the utility of defining transitions not on the basis of a fixed time interval, as is characteristic of a true FMC process, but rather by treating a change in the algebraic sign of a given component as the criterion for defining transitions from one state to another. This has the effect of introducing varying dwell times that accompany any particular transition.

### Considerations impacting applied definitions of Hb states

While our effort here has been to emphasize quantities that are easily computed, we recognize that more granular descriptions can be obtained by taking into account either the history of state transitions beyond the immediate predecessor, or by imposing additional criteria such as the magnitudes of the Hb-components prior to a transition [26]. While the latter is not currently a criterion for defining states, it is nevertheless considered at a component level, in the measures of flux. Expansion of the state definitions would, however, involve a tradeoff in signal-to-noise, as the fraction of data comprising a given state will be reduced. In practice, for a given application, we would expect that more optimal assignments can be accomplished by adjusting the criteria that are used to define a state, for example by reducing number of Hb components (e.g., collapsing states 3 & 8 into 2 & 7), or, as suggested, by accounting for features of transition history and component amplitudes. These understandings hold whether the primary measures are limited to the sensor space or include additional processing by tomographic reconstruction.

Another consequence of our state definitions has been to limit dimensionality by averaging values of the transition matrix coefficients over time and space. Being thus independent of the pre-transition dwell time, and where in space and when in time the transition occurs, the derived values represent behaviors typical of the entire breast. Thus the methodology purposefully considered a more coarse representation than was required, but one that we believe is more in line with the expected spatial limits of the NIRS technique while also being aligned with the spatial scale on which feedback mechanisms are believed to operate. Also, and because our aim has been to evaluate potential differences among the various coefficient values on a group level, in addition to applying the preceding criteria, evidence for such differences was determined by computing the average response across all subjects in a particular group, separately for the left and right breasts.

Separately, which data values are included in a given state will be sensitive to preprocessing schemes that may serve to smooth or otherwise alter trends in the primary data. Here we have limited preprocessing steps to linear detrending, in recognition that long-term drift is likely instrument related. Not employed have been efforts to eliminate specific intrinsic classes of frequency structure (*e*.*g*., respiratory or cardiac components), whose examination may prove informative.

Yet another factor influencing the quality of information obtained is the composition of the subject groups. Two aged-matched groups were considered: women with confirmed breast cancer in either breast, and those who have other forms of breast disease or have no known breast pathology. Each group comprised subjects whose breast-cup size ranged from B to DD. Thus we can conclude that the derived coefficients are representative of behaviors that have been substantially averaged across multiple potentially confounding factors. As evidenced by the described Results, the resultant formalism supports assignment of a broad class of coefficients, which yield feature patterns that appear mainly independent of each other and are disease-sensitive. Further, and especially gratifying, is the additional evidence that principal features of these coefficient spaces appear to follow simple relationships that are aligned with well-defined principles of chemical reaction theory.

While the methodology put forward is an extension of prior reports by our group [18,24], for completeness and clarity we recognize a superficially similar methodology that has more recently been described [25,26]. The methodologies advanced in the latter reports also evaluate co-varying elements of the Hb signal. Different, however, is the temporal span of measures from which coefficient values are determined and the underlying theory applied to generate them. In these reports, coefficient values are based on instantaneous measures of a time-evolving process [36]. In contrast, because we believe that the underlying processes that drives feedback mechanisms operate as a network with a spatially and temporally varying matrix of transition time constants, the coefficient values that we compute is based on processes that exhibit varying dwell times. Also different is the fundamental theory governing feature generation. Our methodology is motivated by an understanding of stochastic networks, in recognition that feedback behaviors affect not one, but many factors that interact. Recognizing that instantaneous values of co-varying measures trace a trajectory in (ΔdeoxyHb,ΔoxyHb)-space (Fig 1, and its equivalents in the cited literature), Ref. 36 has sought to apply principles of the physics of moving bodies as a strategy for exploring features of those trajectories. While it is not necessary for an applied mathematical formulation to have a strong biological correspondence, arbitrary adoption of laws that are relevant to one field but not applicable to another risks introduction of reification fallacies.

We are aware that the biological interpretation of features derived from data-driven methods can be challenging. This can become important in situations where the goal is not just to classify data, but to deduce meaning from the derived measures as a guide to clinical intervention. We also note that the data transform kernel applied here is a simple counting scheme and does not involve the use of complex operations in the time or frequency domain. We therefore believe that, in contrast to other strategies that might be considered, (*e*.*g*., various temporal decomposition methods), the methodology applied here seems unlikely to distort the underlying biological interpretation of the various state classifications. As noted, while we have presented these within a context that we believe substantially reflects a gradient in oxygen supply-demand balance, substitution of the described sequence by others will not influence the information content, because the data transformations used here are independent of the state labels.

### Implications of applied methodology for application development

Two principal findings documented in this report are: (1) the applied scheme supports identification of a dense coefficient space; (2) the feature patterns seen among the various coefficients appear mainly independent of each other and are disease-sensitive. Normally, evidence of independent features having such sensitivity provides an excellent launching point for optimizing interpretive strategies. These can include the goal of detecting disease or discriminating one type of disease from another, or of appreciating responses to various types of therapeutic interventions [37]. Specific methods well suited to leverage these features are various forms of machine-learning algorithms [38].

Because NIRS measures can be applied to almost any tissue type and subject group, generation of similar coefficient classes from these measures seems feasible. Also recognized is the understanding that there are multiple forms of acute and chronic conditions that can lead to altered tissue perfusion. Among the former are conditions such as wound healing, compartment syndrome, stroke, and other causes of hypoperfused states, while the latter includes diseases whose morbidities can be traced to either vasculo-occlusive disease (*e*.*g*., diabetes) or to sustained inflammatory responses with or without associated vascular malformations (*e*.*g*., cancer, various forms of autoimmune disease). Further, growing evidence that fNIRS measures can be reliably acquired under a wide range of conditions, including unconstrained environments [39,40], adds to the expectation that the described methods could serve to advance applications in these environments as well.

We also note that our demonstration of promising disease detection under resting-state conditions likely holds importance in clinical environments, where constraints commonly arise; for example, subjects may be anesthetized or unconscious for other reasons. The potential for use in outpatient settings would also appear favorable, as the resting-state capability simplifies patient setup requirements.

Further, while our aim here has been to emphasize the practicality of resting-state measures, extension of described methods to neurobehavioral assessments, including sleep studies, appears feasible. The granularity of accessible features could serve to advance biofeedback or brain-computer interface applications. In recognition that connectivity patterns of the brain are not static, a particular focus would be to acquire independent measures of such states while fine-tuning task recognition responses to this evolving background. The recent demonstration that communication with “totally locked-in” subjects can be accomplished from fNIRS measures emphasizes the merits to extend this capability [41].

Separately, the considered methods may have significant preclinical value. Of interest here are the many classes of manipulations intentionally imposed on animal models in pursuit of new drugs or other disease treatments, or to extend basic understandings of the systemic dependences that accompany these manipulations. For instance, much effort is devoted to establishing animal models that have specific disturbances in gene expression, immune response, or properties of the microbiome. While a range of toxicity measures and measures of principal organ function is currently established [42,43], mainly lacking are tools that can evaluate the impacts of these manipulations on the information domain of oxygen delivery to tissue in the intact animal, especially in the form of a non-invasive, high-throughput screening method. Appreciation of the importance of this information domain with regard to overall health, and the observation that many classes of drugs that meet the criteria for human trials go on to fail because of unanticipated side effects, suggest that added attention to this basic feature of tissue function may be warranted.

Further, it is also understood that other sensing strategies intended to explore the time-varying features of the hemoglobin signal could benefit from the methods described here. These would include photoacoustic imaging [44] and new holographic methods that support suppression of the influence of light scattering on image quality [45].

### Future considerations

Access to a dense feature space of substantially independent coefficients may support development of new approaches to computational modeling or new analysis strategies [46]. This brings to mind a more basic understanding of factors contributing to information transduction. For instance, discrete-valued information such as sequences of Hb states can be formally regarded as arbitrary-length “words” in a language having a 10-“letter” alphabet. The same concept applies equally well to sequences of transitions between the current state and its predecessor (for a 90-letter language), the current state and last two predecessors (810 letters), *etc*. The structures and constraints that may be concealed in these sequences are unknown *a priori*, and it is prudent to adopt analysis strategies that can handle arbitrary degrees and types of complexity. Work in other fields has shown that formal language theory (FLT) is well-suited for the type of situations that we are faced with [47]. While this framework was originally intended to appreciate syntactic structures present in human languages, it has proven effective for evaluating a wide range of generative rule-based systems, including computer programs, RNA structure, animal vocalizations, and even music and dance [48,49].

An example of a goal that is conceptually straightforward and supports application of FLT to the methodology defined here, would be determine the level in the Chomsky hierarchy corresponding to a state- or transition-based language, or to derive an appropriate set of grammatical rules [48]. Here we observe that the alphabet that corresponds to the derived language can arise from two distinct representations. Already noted is the assignment of the Hb-states to the letters of an alphabet, with words of arbitrary length. Alternatively, the combinations of Hb-signal component and algebraic sign (*i*.*e*., Δ*D*(-), Δ*D*(+), Δ*O*(-), etc.) may be taken as the ten letters of a language comprising exactly ten words (assuming no additional state definitions are employed), each being five letters long, that may be arranged in any sequence into sentences of any length.

What we find intriguing about the latter representation is its formal similarity to the structure of DNA and RNA, each of which comprises just four chemically distinct bases (“letters”) arranged in groupings of three (“words”), sequences (“sentences”) of which encode a vast quantity of information. As previously discussed [50,51], information of this type can be represented in the context of FLT to provide for useful guidance in the design of RNA molecules. By analogy, the sequence of state transitions that arises from the similarly limited alphabet proposed here may also encode a wide range of information regarding the feedback mechanisms that operate in the resting state.

Practical implementations of this line of inquiry can include the goal of determining the hierarchy level or the number or specific content of grammatical rules, and how these vary across different subject groups. Examples of criteria that may be used for defining these groups include the presence or absence of pathology, different levels of distraction (brain studies), or fatigue or stress in cases where the aim is to appreciate factors affecting overall performance in high-stress environments.

However, the type of undertaking suggested here is more challenging than the sorts of biological questions that typically are addressed using FLT-based methods [48]. The reason is that at least two considerations render the solution non-unique: first, when the information one has about a language consists of a finite number of letter sequences, then grammatical structures able to derive them all can be found at any hierarchical level, provided that no limit is placed on the number of rules [48,52]; second, even if the hierarchical level is specified in advance, typically it is possible to determine multiple sets of grammatical rules that can derive all of the available sequences [52]. Accordingly, a strategy that incorporates multiple criteria is required to decide which, among many combinations of hierarchy level and grammatical-rule sets, is optimal. For example, the goal could be to simultaneously minimize the required hierarchical level and the required number of grammatical rules. (This approach is suggested in analogy to what is done in dynamic causal modeling computations to select the “best” model of effective connectivity from among many hypotheses: there, the algorithm strikes a balance between high goodness-of-fit to experimental data and low model complexity [53].) We also would anticipate making extensive use of cross-validation techniques [54], to test for overfitting and to evaluate the sensitivity of different combinations of hierarchical level and grammatical rules to the order of data presentation.

The preceding suggestion constitutes a particular approach to increasing the type and quantity of information that could be extracted from accessible features of fNIRS time-series data. In parallel, we have begun to explore methods for expressing those features in terms of other quantities that have biological relevance but are not directly observable.

Thus we acknowledge the potential to extend the network representation considered here into one where the goal is to derive coefficients from a hidden network of coefficients that reflect features corresponding to details of feedback mechanisms.

To make this idea less abstract, we briefly describe one plausible modeling scheme that might be considered as a first-order representation of the state transition network. In particular, we consider the framework of a DC electrical network [55], in recognition of the analogies that can be drawn between *P*_*fi*_ (or *P*_*fi*_*ϕ*_*X*_(*f*,*i*) = *m*_*fi*_) and *k*_*fi*_ to the electrical current and conductance (*i*.*e*., 1/resistance) represented in Ohm’s Law. Connection to the physiological processes underlying observed patterns of state transition coefficients comes from an understanding that real-world electric-circuit networks are typically based on a distribution of power sources required to drive various specialized functionalities. As our Hb-state representation considers behaviors that originate on a macroscopic scale within an inhomogeneous underlying tissue architecture, it seems likely that the factors that modulate the Hb signal will also influence features of the state network in a distributed manner. Consequently, our attention in formulating a network representation has been to devise a scheme that allows for derivation of coefficients that correspond to such distributions.

The definitions of *k*_*fi*_, *P*_*fi*_ and *ϕ*_*X*_(*f*,*i*) suggest a correspondence between *k*_*fi*_ and electrical conductance, and likewise between either *P*_*fi*_ or *P*_*fi*_*ϕ*_*X*_(*f*,*i*) and current. (The *P*_*fi*_ values are “transition currents” if transitions are analogized to a type of particle, while *P*_*fi*_*ϕ*_*X*_(*f*,*i*) values are concentration-change currents or saturation-change currents.) The different Hb states can be likened to nodes in an electrical network, and the quantity 1/*k*_*fi*_ to the value of a resistor that conducts current from the *i*^th^ node to the *f*^th^ (more strictly, a resistor in series with an ideal diode, to allow for *k*_*fi*_ ≠ *k*_*if*_). These correspondences bring to mind at least two additional analogies to properties of electrical circuits.

1. A new quantity corresponding to voltage can be defined, by applying an analogue of Ohm’s law to the transition-probability and rate values:
  $$V_{TC}\left(f,i\right)=\frac{k_{fi}}{P_{fi}},\;V_{X}\left(f,i\right)=\frac{k_{fi}\phi_{X}\left(f,i\right)}{P_{fi}},$$ (14)
  where *X* is any of the Hb-signal components *D*, *E*, *O*, *S* or *T* in Eq (5).
2. In the typical framing of electrical-network analysis problems [56], the resistances and applied electromotive forces (EMFs) are known quantities and the voltages and currents are the unknowns that are solved for. A different, inverse, version of the problem is suggested here, in that the currents and resistances are known quantities, and what must be found is an EMF or combination of EMFs that would produce the known currents, given the known structural (connectivity and resistances) and dynamic (currents) properties of the network. Assuming these quantities can be identified with a degree of confidence, then the considered approach would seem capable of amplifying our understanding of the actions of feedback mechanisms whose influences can be directly observed but whose details remain hidden.

As a separate avenue for potential future developments, we also recognize the potential to express properties of the defined states in terms of their sensitivities to more primitive behaviors. Here we refer to a methodology recently described by our group, which demonstrated that cross-domain moments of spatiotemporal behaviors (*i*.*e*., mean, variance) can be distinguished in terms of their sensitivity to these primitive behaviors [16]. While our emphasis in this report has been to explore features of data that undergo specific state transitions, we also recognize that the cloud of points corresponding to each Hb state (*e*.*g*., see Fig 3), can be represented as a time series. In fact, because each state is a composite of the five elements of the Hb signal, five time series can be defined for each state. For each of these it can be shown that there are fourteen nontrivial cross-domain (space-time) moments that can be defined (*i*.*e*., the eight that are considered in Ref. 16 plus an additional six, five of which would be trivial (because always exactly zero) should the Hb signal not be separated into states). Our interest in these quantities is motivated by the expectation that as the underlying drivers of tissue-vascular coupling vary (*e*.*g*., as they are influenced by actions of disease), they can imprint different spatiotemporal behaviors that are sensitive to the indicated primitives. Thus we acknowledge that in addition to the above-described transition-dependent network properties, representations of bulk features of the network may add to the differential information that is accessible.

To summarize, in this report we have demonstrated the influences that intrinsic feedback mechanisms have on the matrix of coefficient values that are accessible from a finite-state representation of co-varying elements of the hemoglobin signal explored under resting-state conditions, for affected and unaffected breasts. Evidence obtained strongly indicates that the influence of disease differs significantly across the various feature spaces.

The molecular biology and neurosensing communities have long recognized that the information content of macromolecular structures (*e*.*g*., DNA, RNA, proteins) and the network of neural signaling that occurs in the brain, respectively, constitute biological languages [49,57,58]. Both classes of information follow a set of rules (*i*.*e*., grammar) and a hierarchy of relationships among these rules in ways that are similar to rules of linguistics. Thus we consider features of the Hb signal identifiable from our methods as constituting “conversations” between the vasculature and its host tissue that are conveyed in the “language of hemodynamics.” Also, whether this understanding and other suggested methods actually prove to offer useful benefits or not, we are still left with our explicit demonstration that the identified features have a substantial independence in their sensitivity to a representative disease type (cancer) for a representative tissue class (breast). Demonstration of this previously unrecognized independence should support new opportunities to appreciate common features of elementary factors (*e*.*g*., its rules) that are often expressed by the information rich domains of biological systems [59].

Complex phenomenology in dynamical systems often can be reduced to more simplified descriptions, provided that the rules governing state-space behaviors can be determined [60]. Evidence that the identified features are accessible under easily implemented experimental conditions (*i*.*e*., the resting-state) likely serves to enhance development of practical uses.

## Supporting information

S1 Appendix Hb signal-component axes in Fig 1. Proof that orientations of the ΔtotalHb axis, ΔHbO_2_Exc axis, and ΔHbO_2_Sat demarcator in Fig 1 are uniquely determined by the mathematical definitions of these dependent quantities [includes Supporting Information Figs A, B and C].
(DOCX)

S2 Appendix Relevant understandings from stochastic network theory. Comparison of features of applied finite-state model and a finite Markov chain.
(DOCX)

S3 Appendix Relationship of (continuous) state-space trajectories to finite-state representation. A graphical explanation of the scoring methods used in computing coefficients such as the transition probability and transition rate constant [includes Supporting Information Figs D and E].
(DOCX)

S4 Appendix Coupled behaviors of state transitions. Description of physiological factors considered likely to constrain permissible relationships among selected classes of state transitions [includes Supporting Information Fig F].
(DOCX)

S5 Appendix Informative trends among Hb-component coefficient values. Replots of transition intrinsic-flux measures, to highlight trends that are otherwise difficult to appreciate [includes Supporting Information Figs G and H].
(DOCX)
