# Supplementary material for: Hemoglobin state-flux: A finite-state model representation of the hemoglobin signal for evaluation of the resting state and the influence of disease
Source: PLoS One. 2018 Jun 8;13(6):e0198210. doi: 10.1371/journal.pone.0198210 (PMC5993307; doi:10.1371/journal.pone.0198210)
Supplement: S1 Appendix — Proof that orientations of the ΔtotalHb axis, ΔHbO2Exc axis, and ΔHbO2Sat demarcator in Fig 1 are uniquely determined by the mathematical definitions of these dependent quantities [includes Supporting Information Figs A, B and C]. (DOCX) [file pone.0198210.s001.docx]

**Hb Signal-Component Axes in Fig 1**

When ΔdeoxyHb and ΔoxyHb are graphically depicted as perpendicular axes in a two-dimensional plot, the orientations of the ΔtotalHb axis, ΔHbO_2_Exc axis, and ΔHbO_2_Sat demarcator are uniquely determined by the mathematical definitions of these dependent quantities. Here we show that the orientations assigned in Fig 1 correspond to those definitions.

Each axis in a conventional two-dimensional Cartesian plot (*i.e.*, an “*x*-*y* plot”) has three properties that are illustrated in Fig A(a) for the x-axis and in Fig A(b) for the y-axis. The *x*-axis (*y*-axis) passes through the origin of the coordinate system; it is perpendicular to every line defined by an equation of the form *x* =*k* (*y* =*k*), where *k* is a constant; and it separates the *y* > 0 and *y* < 0 (*x* > 0 and *x* < 0) regions of the plane. The ΔdeoxyHb (Δ*D*) and ΔoxyHb (Δ*O*) axes of Fig 1 are a particular example of the general case depicted in Fig A, and consequently have the same three properties. We shall determine the locations and orientations for the ΔtotalHb (Δ*T*) and ΔHbO_2_Exc (Δ*E*) axes by requiring them to have the same properties, with respect to these two Hb-signal components.

**Fig A. Defining properties of the (a) *x*-axis and (b) *y*-axis in a 2D Cartesian plot.**


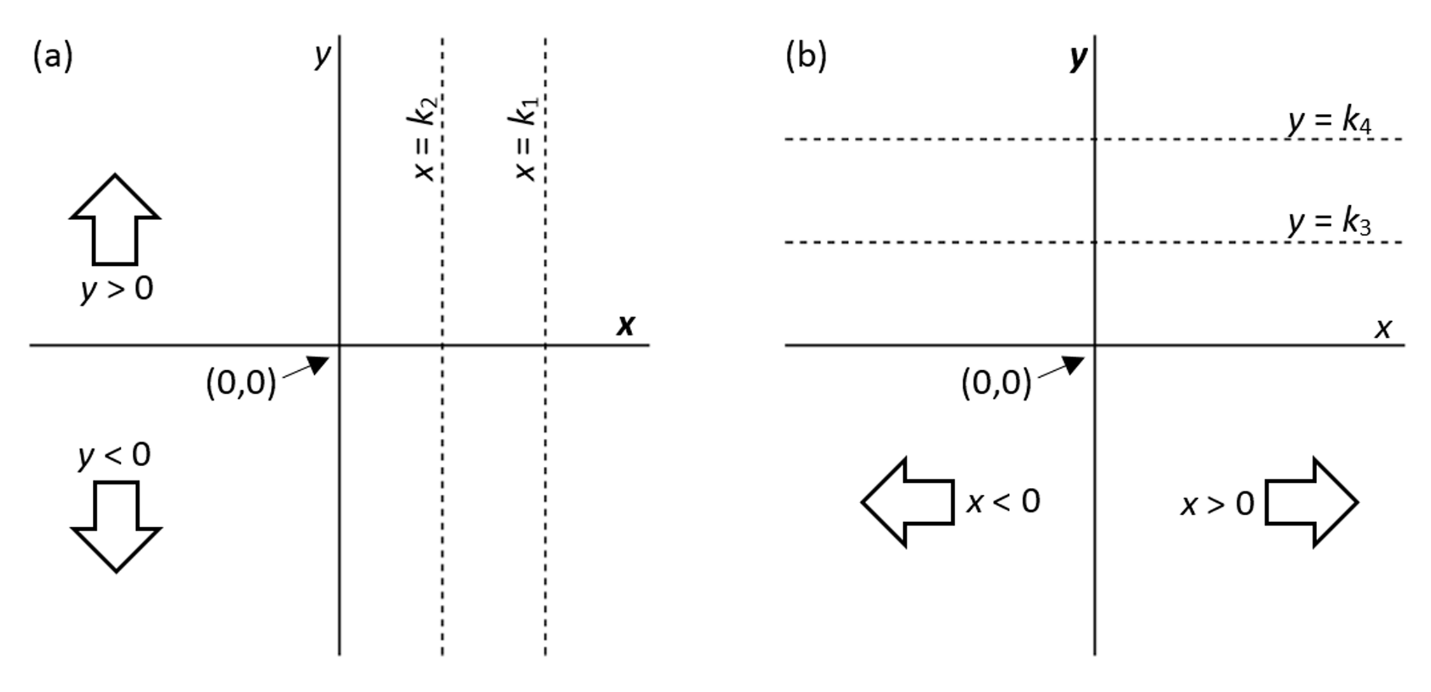


(a) The *x*-axis separates the plane into regions *y* < 0 and *y* > 0, and is orthogonal to all lines of constant *x*; (b) the *y*-axis separates the plane into regions of *x* < 0 and *x* > 0, and is orthogonal to all lines of constant *y*. Both axes pass through the origin of the coordinate system.

Given the definition of Δ*T* in terms of Δ*D* and Δ*O*, constraining Δ*T* to a constant value enforces a specific relationship between Δ*D* and Δ*O*:

$$\begin{matrix} \Delta T=\Delta D+\Delta O=k \\ \Delta O=k-\Delta D, \end{matrix}$$

which shows that any function of constant Δ*T* is a straight line with slope of -1. The analogous derivation for Δ*E* is

$$\begin{matrix} \Delta E=\Delta D-\Delta O=k \\ \Delta O=\Delta D-k, \end{matrix}$$

from which it follows that any function of constant Δ*E* is a straight line with slope of +1. The Δ*T* axis is, by definition, orthogonal to all the constant-Δ*T* lines, and this requirement is met only by the line that passes through the origin (another definitional requirement) and is rotated 45° to the positive Δ*D* axis, as illustrated in Fig B(a). Similarly, the Δ*E* axis is orthogonal to all constant-Δ*E* lines, and only the line that passes through the origin and is rotated 135° to the positive Δ*D* axis, as shown in Fig B(b), satisfies this requirement. Inspection of Fig B further shows that the Δ*T* and Δ*E* axes fulfill the third definitional property, in that the Δ*T* (Δ*E*) axis separates the plane into Δ*E* > 0 and Δ*E* < 0 (Δ*T* > 0 and Δ*T* < 0) regions. Note also that the algebraic signs of the intercepts in Eqs. and determine which half-planes are positive and which are negative.

**Fig B. Defining properties of the (a) ΔtotalHb (Δ*T*) axis and (b) ΔHbO_2_Exc (Δ*E*) axis in the Fig 1 coordinate system.**


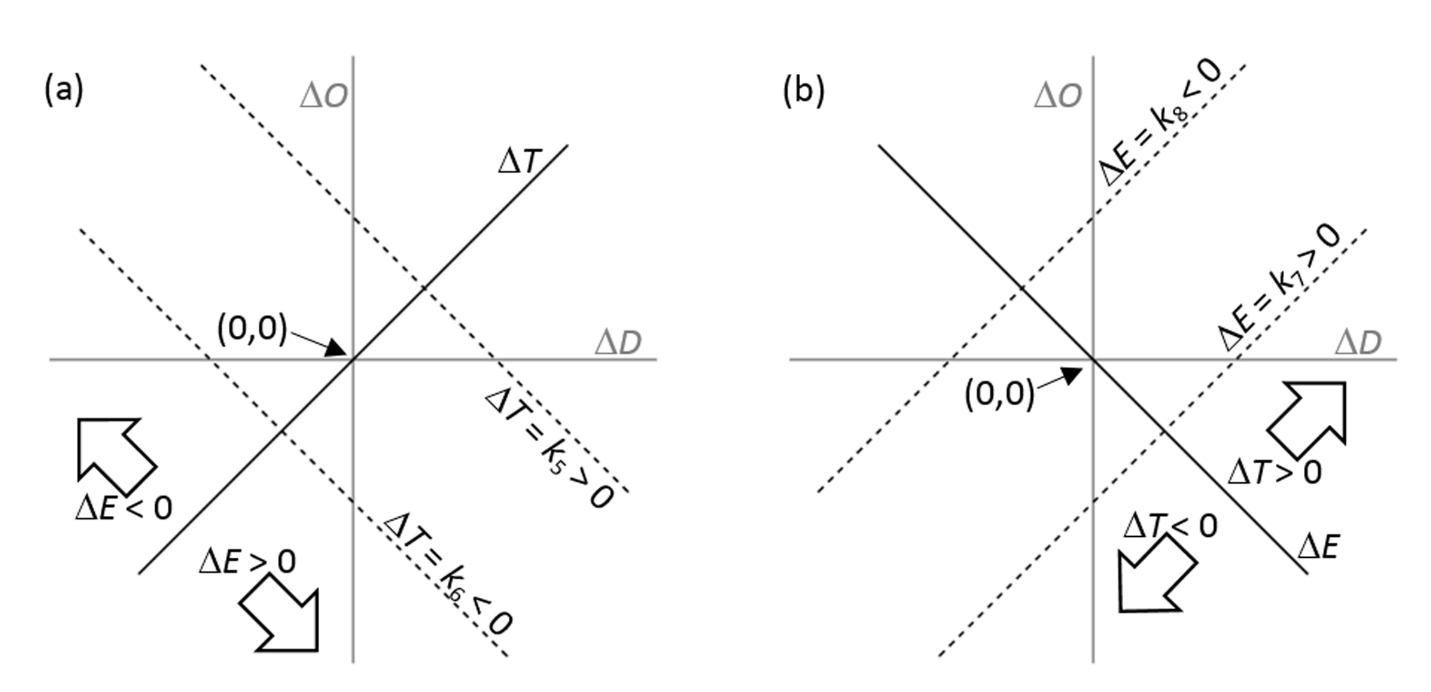


The black solid lines have the three properties indicated in Fig A, with respect to the Δ*T* and Δ*E* Hb signal components. Therefore, the black solid lines in Fig B(a) and B(b) are the Δ*T* and Δ*E* axes, respectively.

Because there is an odd number of Hb-signal components overall, and ΔHbO_2_Sat (Δ*S*) is the component that is not paired with another, there is a noteworthy difference between the criteria we use to determine its “axis” and those of the other four. In particular, the Δ*S* demarcation line is defined as the function that separates the Fig 1 plane into Δ*S* > 0 and Δ*S* < 0 regions (*i.e.*, it is Δ*S* = 0). In determining that this is a straight line passing through the origin, as indicated in Fig 1, we must account for the fact that, in contrast to the other components, Δ*S* depends on the time-averaged absolute deoxyHb and oxyHb concentrations (*D*_0_ and *O*_0_, respectively), in addition to the fluctuations about those average values. That is:

$$S_{0}=100\frac{O_{0}}{D_{0}+O_{0}},$$

and

$$S=S_{0}+\Delta S=100\frac{O_{0}+\Delta O}{D_{0}+O_{0}+\Delta D+\Delta O}.$$

Therefore,

$$\begin{matrix} \Delta S=S-S_{0}=100\left(\frac{O_{0}+\Delta O}{D_{0}+O_{0}+\Delta D+\Delta O}-\frac{O_{0}}{D_{0}+O_{0}}\right) \\ =100\frac{\left(D_{0}+O_{0}\right)\left(O_{0}+\Delta O\right)-O_{0}\left(D_{0}+O_{0}+\Delta D+\Delta O\right)}{\left(D_{0}+O_{0}\right)\left(D_{0}+O_{0}+\Delta D+\Delta O\right)} \\ =100\frac{O_{0}\left(D_{0}+O_{0}\right)+D_{0}\Delta O+O_{0}\Delta O-O_{0}\left(D_{0}+O_{0}\right)-O_{0}\Delta D-O_{0}\Delta O}{\left(D_{0}+O_{0}\right)\left[\left(D_{0}+O_{0}\right)+\Delta D+\Delta O\right]} \\ =100\frac{D_{0}\Delta O-O_{0}\Delta D}{\left(D_{0}+O_{0}\right)^{2}+\left(D_{0}+O_{0}\right)\Delta D+\left(D_{0}+O_{0}\right)\Delta O}. \end{matrix}$$

The algebraic sign of Δ*S* is the same as that of the numerator of the ratio in Eq. (because the denominator is the product of two non-negative terms). Consequently, if our only goal were to confirm that the Δ*S* demarcator depicted in Fig 1 is correct, it would be sufficient to set the numerator equal to zero and solve for Δ*O*:

$$\begin{matrix} D_{0}\Delta O-O_{0}\Delta D=0 \\ \Delta O=\frac{O_{0}}{D_{0}}\Delta D. \end{matrix}$$

The result is a straight line passing through the origin, with slope of *O*_0_/*D*_0_. That this ratio is equal to the expression given in Methods: Relationships among co-varying elements of the Hb signal follows from:

$$\begin{matrix} \frac{O_{0}}{D_{0}}=\frac{O_{0}}{T_{0}-O_{0}}=\left(\frac{T_{0}-O_{0}}{O_{0}}\right)^{-1}=\left(\frac{T_{0}}{O_{0}}-1\right)^{-1}=\left[\left(\frac{O_{0}}{T_{0}}\right)^{-1}-1\right]^{-1} \\ =\left[\left(\frac{S_{0}}{100}\right)^{-1}-1\right]^{-1}=\left(\frac{100}{S_{0}}-1\right)^{-1}=\left(\frac{100-S_{0}}{S_{0}}\right)^{-1}=\frac{S_{0}}{100-S_{0}}, \end{matrix}$$

and a line with slope *S*_0_/(100 – *S*_0_) is inclined at an angle of tan^-1^[*S*_0_/(100 – *S*_0_)] to the positive Δ*D* axis.

While the preceding analysis is sufficient for the purpose of deriving the Δ*S* demarcator in Fig 1, it can be extended to address a question that arises when considering that the other four components occur in conjugate pairs (*i.e.*, either Δ*E* and Δ*T* axes, or Δ*D* and Δ*O*, can serve as coordinates for specifying the location of any point in the plane), and HbO_2_Sat does not. For completeness, it is instructive to show that a quantity forming a conjugate pair with Δ*S* can be mathematically defined, but that it is not biologically meaningful. To accomplish this, we proceed as in the previous cases and determine what is the function corresponding to a fixed value of Δ*S*, Δ*S* = *k*.

$$\begin{matrix} \Delta S=100\frac{D_{0}\Delta O-O_{0}\Delta D}{\left(D_{0}+O_{0}\right)^{2}+\left(D_{0}+O_{0}\right)\Delta D+\left(D_{0}+O_{0}\right)\Delta O}=k \\ 100D_{0}\Delta O-100O_{0}\Delta D=k\left(D_{0}+O_{0}\right)^{2}+k\left(D_{0}+O_{0}\right)\Delta D+k\left(D_{0}+O_{0}\right)\Delta O \\ 100D_{0}\Delta O-k\left(D_{0}+O_{0}\right)\Delta O=100O_{0}\Delta D+k\left(D_{0}+O_{0}\right)^{2}+k\left(D_{0}+O_{0}\right)\Delta D \\ \left[\left(100-k\right)D_{0}-kO_{0}\right]\Delta O=\left[kD_{0}+\left(100+k\right)O_{0}\right]\Delta D+k\left(D_{0}+O_{0}\right)^{2} \\ \Delta O=\frac{kD_{0}+\left(100+k\right)O_{0}}{\left(100-k\right)D_{0}-kO_{0}}\Delta D+\frac{k\left(D_{0}+O_{0}\right)^{2}}{\left(100-k\right)D_{0}-kO_{0}}. \end{matrix}$$

As was the case for the other four components, each Δ*S* = *k* function is a straight line in the (Δ*D*,Δ*O*) coordinate system. However, Δ*S* is unique in that the slope of the line is a function of *k*. This requires that the lines corresponding to any two distinct values of *k* have an intersection point, which raises the question of how lines corresponding to different fixed values of Δ*S* can have a point in common. The only possible resolution is for the intersection to occur at the origin of the absolute-concentration coordinate system (*D* and *O* axes) that is sketched in Fig C. That this is in fact where the intersection falls can be seen by setting Δ*D* equal to –*D*_0_ in the last line of Eq. , then rearranging the equation to obtain Δ*O*(–*D*_0_) = –*O*_0_.

**Fig C. Lines of constant Δ*S*, and an associated orthogonal trajectory.**


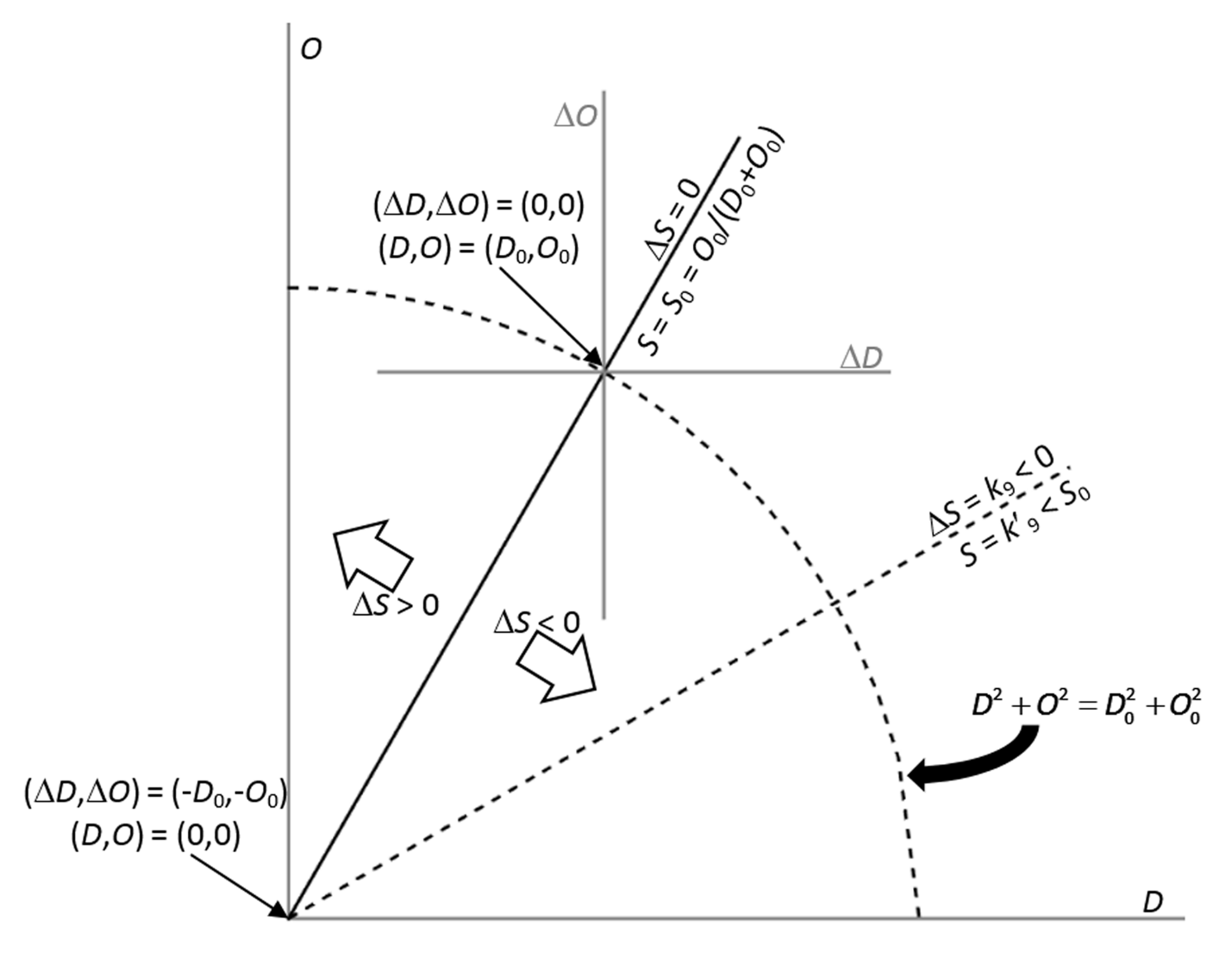


Also identified is the relationship between absolute-concentration (*D*,*O*) and concentration-change (Δ*D*,Δ*O*) coordinate systems. *D*, *O* = absolute-concentration variables; *D*_0_, *O*_0_ = time-averaged, fixed values of the deoxyHb and oxyHb concentrations, respectively, for a given dataset; Δ*D*, Δ*O* = variable concentration changes with respect to the time-averaged concentrations.

Note that in(de)creasing the value of *k* in Eq. (*i.e.*, in(de)creasing HbO_2_Sat) causes an in(de)crease in the slope of the constant-Δ*S* line. This fact determines which regions in Fig C correspond to Δ*S* > 0 and to Δ*S* < 0.

Finally, we see that the conjugate-to-Δ*S* component does not correspond to a straight line, but to a curve that intersects each constant-Δ*S* curve at an angle of 90° and that passes through the origin of the (Δ*D*, Δ*O*) coordinate system. The constant-Δ*S* curves are a family of straight lines radiating out from (*D*,*O*) = (0,0), and so the orthogonal trajectory that we seek is one quarter (because *D* ≥ 0 and *O* ≥ 0) of a circle, centered at (*D*,*O*) = (0,0), with radius $\sqrt{D_{0}^{2}+O_{0}^{2}}$. While this circular arc may have some utility as a mathematical construct, it is not physiologically meaningful, and we conclude that to treat it as if it does [1] is a fallacy of misplaced concreteness [2,3]. While we have indicated that there might be value in extending the definitions of Hb states to include radial-dimension information (see Methods: Quantification of inter-state Hb-transition coefficients, and Discussion: Considerations impacting applied definition of Hb states), the interpretation provided here indicates that this extension must be undertaken in a biologically informed way if it is to yield a useful finite-state representation. Accordingly, while the finite-states model of this report does make use of information about the magnitudes of the Hb-signal components, it does not do so in the manner that is depicted in Fig C. Instead, it is incorporated in two other ways. The first is that the Hb states are defined in terms of the algebraic signs of all five Hb-signal components, which imposes the quantitative relationships listed in Table 1 (Methods: Relationships among co-varying elements of the Hb signal). The second is by means of the defined intrinsic and weighted flux coefficients (Methods: Transition flux computation, and Methods: Computation of weighted transition fluxes).

**References**

[1] Kato T, inventor. Apparatus for evaluating biological function, a method for evaluating biological function, a living body probe, a living body probe mounting device, a living body probe support device and a living body probe mounting accessory. United States patent US 20080262327. 2013 Mar 26.

[2] Thompson HE. The fallacy of misplaced concreteness: Its importance for critical and creative inquiry. Interchange. 1997;28:219-30.

[3] Prausnitz J. The fallacy of misplaced concreteness. Biophys J. 2015;108:453-4.
