# Supplementary material for: Hemoglobin state-flux: A finite-state model representation of the hemoglobin signal for evaluation of the resting state and the influence of disease
Source: PLoS One. 2018 Jun 8;13(6):e0198210. doi: 10.1371/journal.pone.0198210 (PMC5993307; doi:10.1371/journal.pone.0198210)
Supplement: S2 Appendix — Comparison of features of applied finite-state model and a finite Markov chain. (DOCX) [file pone.0198210.s002.docx]

**Relevant Understandings from Stochastic Network Theory**

Over time, data points corresponding to the instantaneous ΔHb values occupy the ten sectors depicted in Fig 1 in a sequence that is not definitively predictable, is different for different image voxels, and likely is influenced by pathology and other biological factors. To a first approximation, the occupancy of these states, and the transitions among them, can be treated as a finite Markov chain (FMC) in which each sector is a single point or ‘state’ (recognizing that in doing so we discard information regarding distances from the origin and boundaries) [1]. However, while the developed methodology has been strongly motivated by an understanding of FMCs, we have chosen not to impose on the network dynamics the history-independence property (*i.e*., the “Markovian property” [2]) that characterizes a true FMC [1,2]. Instead, the analysis adopted recognizes that the probability for transition to a specified next state may depend on one or more prior states in addition to the current one. It follows that the current method qualitatively differs from the theory of FMCs in two noteworthy ways.

First, it is a consequence of the Markovian property that the *k*-step transition matrix for a time-homogeneous FMC is equal to the *k*^th^ power of the one-step matrix [1]. As our method does not assume the Markovian property (although we do assume that the transition probabilities are time-homogeneous, as the application considered here is to resting-state data), we do not attempt to evaluate powers of the transition matrix, and consequently do not find it necessary to define “step” as a fixed interval of time that is the same for all types of transition. Rather, we define it as the next transition to occur, irrespective of the length of time that a voxel dwells in the pre-transition state. Thus, the *ij*^th^ element of the transition matrix is the probability that the next transition to occur will be from state *j* to state *i*, and not the probability that a transition of the indicated type will occur in the next second (or other time unit). This difference requires us, but also affords us the opportunity, to perform a parallel, independent computation of the average rates for all possible transitions.

Second (and also a consequence of not requiring our mathematical model to have the Markovian property), the elements of the transition-probability matrices we compute are absolute or unconditional probabilities, as opposed to the conditional probabilities that are employed in FMC analyses. Thus our transition-probability matrix contains information that in a conventional FMC computation would reside in two distinct quantities, the (conditional) transition-probability matrix and the so-called fixed vector [1]. But this does not represent a loss of information, as in our application the content of the fixed vector is both independently calculable and extractable from the transition-rate and (unconditional) transition-probability matrices.

**References**

[1] Doyle PG, Snell JL. Random walks and electric networks [Internet]. Available from: <https://math.dartmouth.edu/~doyle/docs/walks/walks.pdf>.

[2] Vrbik J, Vrbik P. Finite Markov Chains. In: Informal Introduction to Stochastic Processes with Maple. New York: Springer; 2013.
