# Supplementary material for: Hemoglobin state-flux: A finite-state model representation of the hemoglobin signal for evaluation of the resting state and the influence of disease
Source: PLoS One. 2018 Jun 8;13(6):e0198210. doi: 10.1371/journal.pone.0198210 (PMC5993307; doi:10.1371/journal.pone.0198210)
Supplement: S3 Appendix — A graphical explanation of the scoring methods used in computing coefficients such as the transition probability and transition rate constant [includes Supporting Information Figs D and E]. (DOCX) [file pone.0198210.s003.docx]

**Relationship of (Continuous) State-Space Trajectories to Finite-State Representation**

To facilitate understanding of the finite-state representation and the derivation of associated metrics, here we present a graphical explanation of the transition scoring methods. As outlined in Methods, the input to our finite-state model is the reconstructed 3D image time series for ΔoxyHb and ΔdeoxyHb. To reduce dimensionality, we treat the ensemble of voxel trajectories in ΔdeoxyHb–ΔoxyHb space as a single data set whose individual elements lie within the coordinate system depicted in Fig 1. As illustrated in Fig 3, a plot of these values produces a dense cloud of points. A reduced example of the same type of data representation is shown in Fig D. Here we have limited the number of voxel trajectories to one, by computing the corresponding spatial mean time series, and have further simplified the graph by plotting only the spatial-mean data values for the first 50 time frames. Note that for ease of viewing, different concentration ranges are plotted on the Fig D coordinate axes, which has the effect of expanding the sectors for some Hb states (*e.g.*, 3, 4, 8 and 9) and compressing others (*e.g.*, 1, 5, 6 and 10). Also to facilitate viewing, different temporal segments of the trajectory have been color coded. Inspection of this plot reveals portions where crossings from one Hb state to another take place between successive time frames, and others that do not.

**Fig D. Plot of reduced time series in ΔdeoxyHb, ΔoxyHb space.**


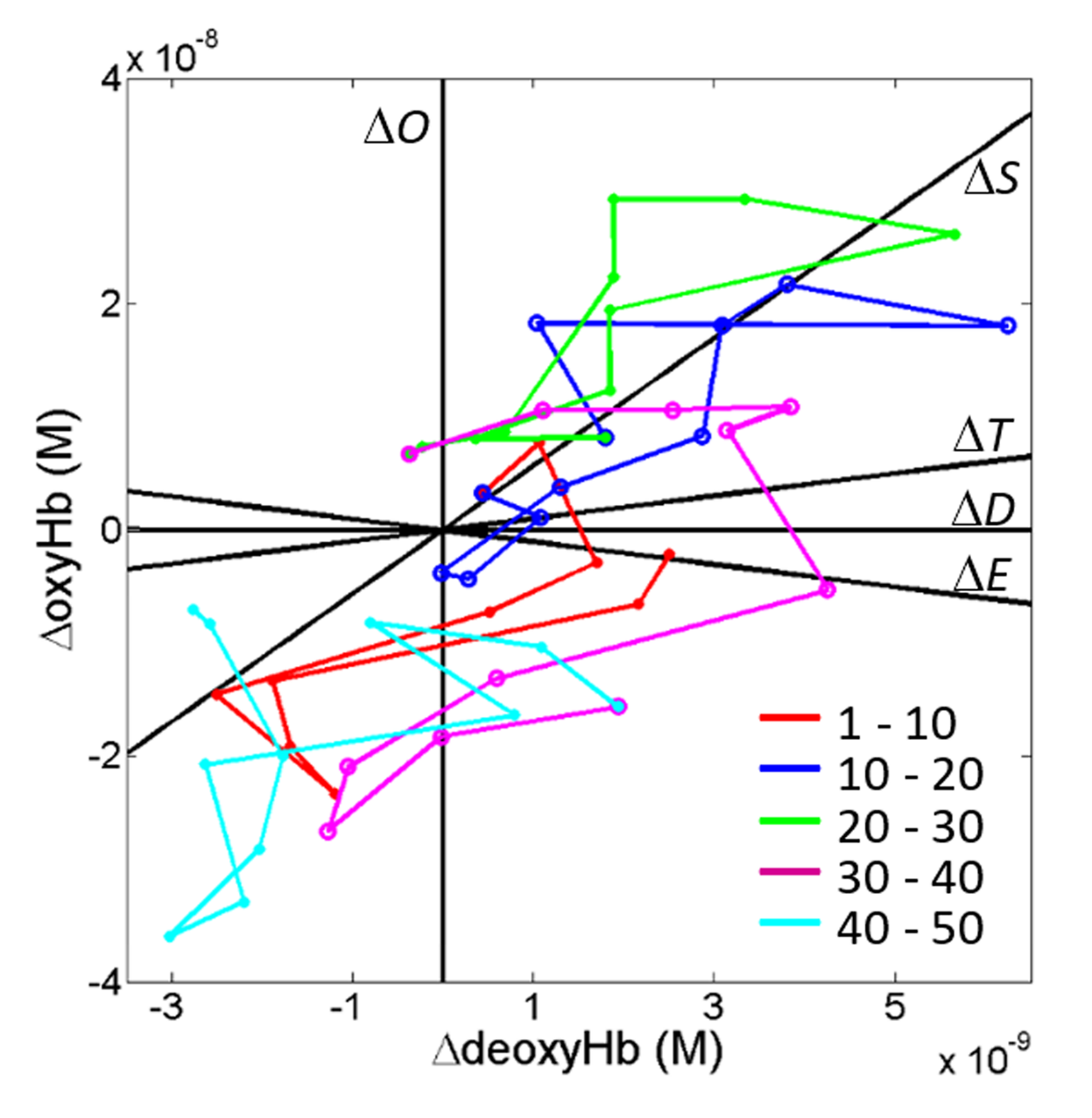


Note some portions of the time series undergo a change in Hb state (*i.e.*, a crossing of one or more components axes), while others dwell in a given state.

In Fig E(a), we have isolated only those portions of the time series in which a transition occurs to a different Hb state upon the next time step. This information is used to compute the transition probability [Methods: Computation of state transition probability, Eq. (1)] and transition flux [Methods: Transition flux computation, Eq. (6)] coefficients. Shown in Fig E(b) is a 9-time frame segment of the time series wherein state transitions occur (red) and others where the trajectory dwells in a given state (blue). This information is used to compute transition rates [Methods: Computation of state transition rate, Eq. (2)].

**Fig E. Subsets of reduced time series relevant to computation of transition coefficients.**


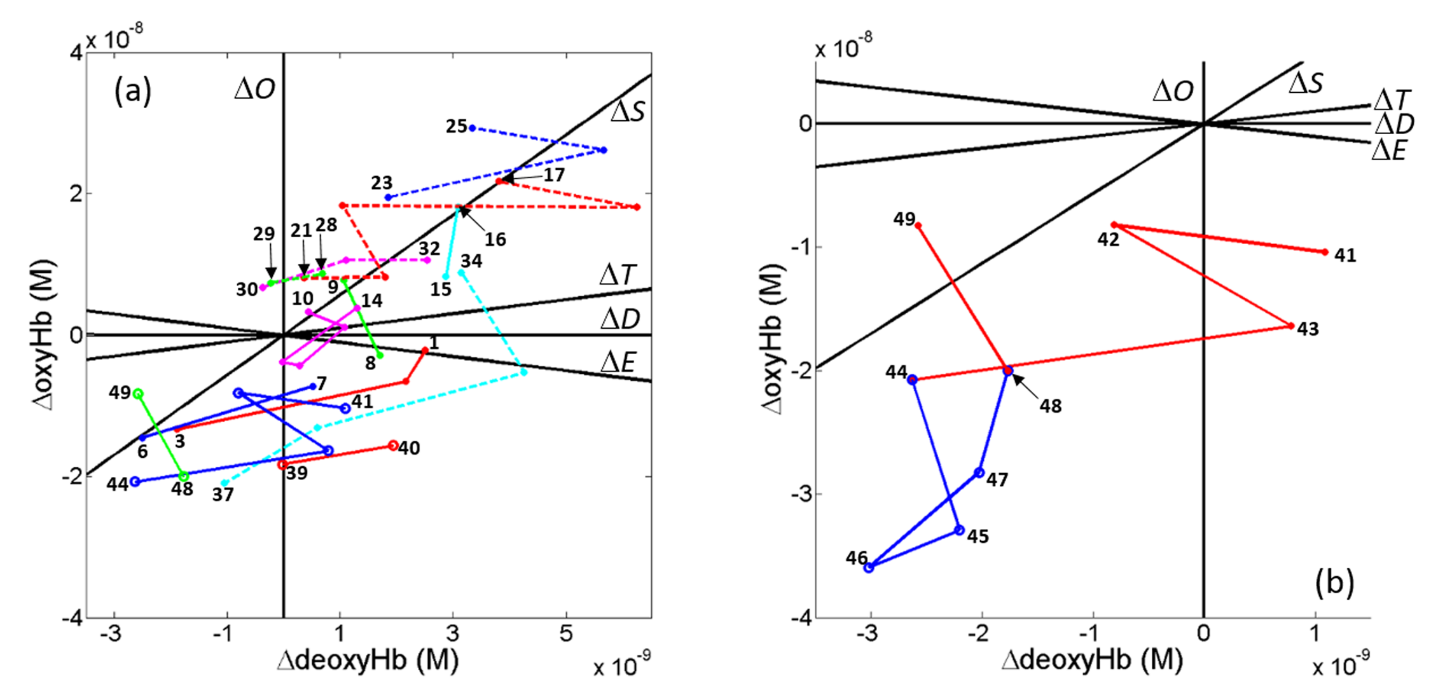


Fig E(a) shows time segments that undergo a state transition upon the next time step. Fig E(b) identifies a segment that, in addition to this behavior (red lines and points), includes a portion that dwells in a given state prior to the next transition (blue). Numbers adjacent to selected data points indicate the associated time frames.

To amplify these understandings we can use the data plotted in Fig E(b) to compute transition rates and probabilities, without having to consider features of signal amplitude (required for computations of transition flux). During the specified time segment we observe three consecutive transitions between States 3 and 4 between the 41^st^ and 44^th^ time frames, followed by a 4 time-step dwell in State 3 and then a transition to State 2. From this we compute a 4→3 transition probability of ½ (because, of the four transitions that occur, two are from State 4 into State 3) and 3→4 and 3→2 probabilities of ¼ each. We likewise compute a transition rate of 1/5 for 3→2 transitions (because the transition from State 3 into State 2 is not completed until 5 time frames after the preceding transition into State 3), and a higher rate of 1 for 3→4 and 4→3 transitions.

The information plotted in Fig E additionally shows that there are differences in detail among the events that are classified as a single type of Hb-state transition. For example, each of the four 7→8 transitions in Fig E(a) begins at a different point in State-7 sector and goes to different point in the State-8 sector. However, the physiological plausibility and demonstrated diagnostic utility of the transition probability and rate coefficients show that this degeneracy is not problematic for the finite-states approach developed in this report. Moreover, the quantitative information that is left out of the probability and rate computations is retained in the computations of intrinsic and weighted fluxes.
