# Supplementary material for: Hemoglobin state-flux: A finite-state model representation of the hemoglobin signal for evaluation of the resting state and the influence of disease
Source: PLoS One. 2018 Jun 8;13(6):e0198210. doi: 10.1371/journal.pone.0198210 (PMC5993307; doi:10.1371/journal.pone.0198210)
Supplement: S4 Appendix — Description of physiological factors considered likely to constrain permissible relationships among selected classes of state transitions [includes Supporting Information Fig F]. (DOCX) [file pone.0198210.s004.docx]

**Coupled Behaviors of State Transitions**

Here we provide a description of the manner in which selected classes of state transitions are influenced by physiological factors that are likely to constrain permissible relationships.

The component axes (black lines) in Fig F are the same coordinate system as in Fig 1, but rotated 45° so that the Δ*E* and Δ*T* axes have the horizontal and vertical orientations, respectively. In this view, the component of any transition vector (colored arrows) parallel to the vertical axis is proportional to the net change in blood volume. The five (out of 90) types of Hb-state transition explicitly depicted are the type that we refer to as “reciprocal” transitions (*i.e.*, the algebraic signs of all five Hb-signal components change), and all are hyperemic. (The other five reciprocal transitions (*e.g.*, 5→10, 9→4), in which blood volume decreases, are not shown.) They are intentionally depicted as all having the same initial and final values for Δ*T*, to emphasize the fact that the principal differences among them lie in the orthogonal, Δ*E*, dimension, which is a measure of the balance between oxygenated and deoxygenated Hb.

**Fig F. Schematic of Hyperemic Reciprocal Hb-State Transitions.**


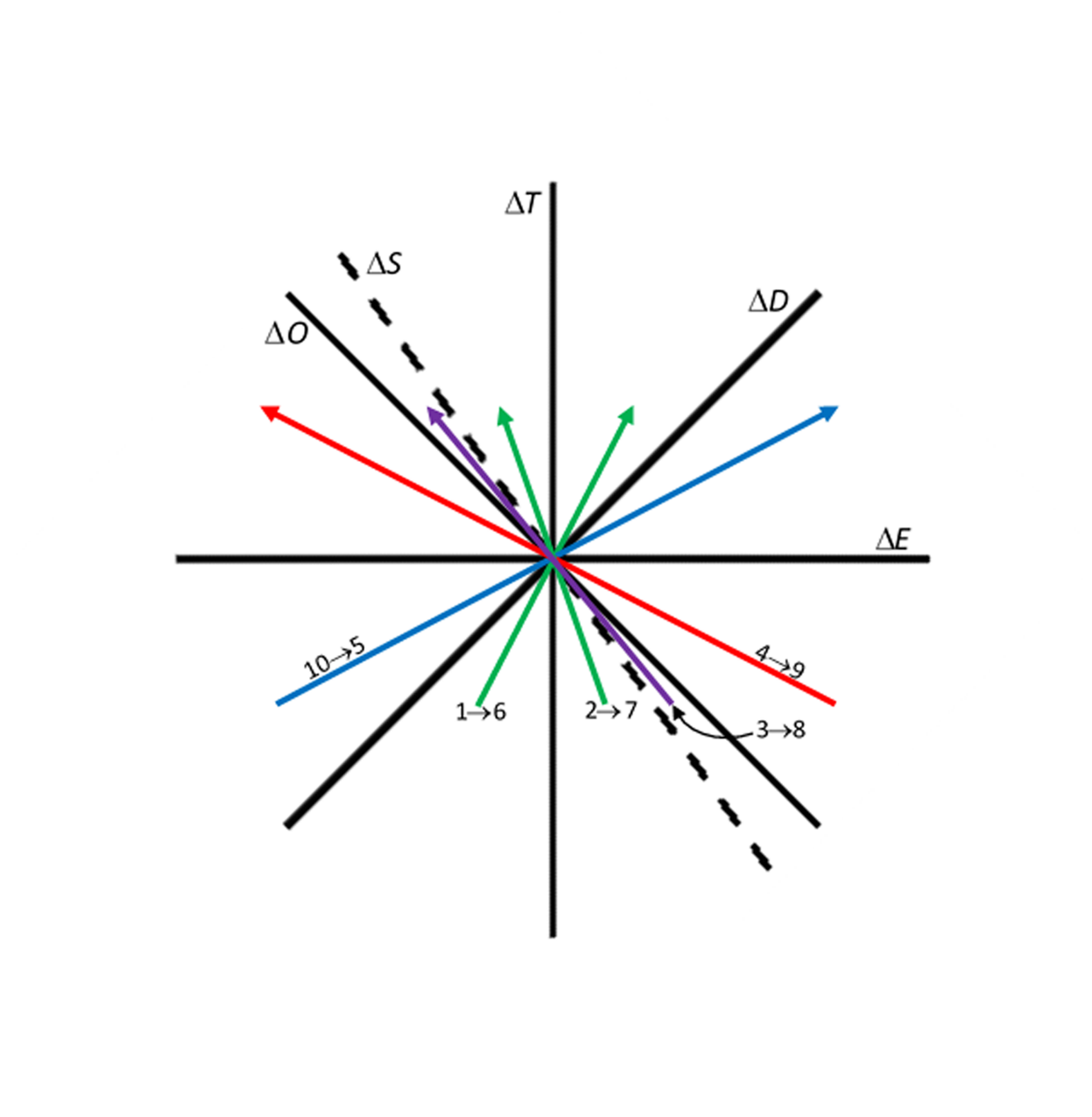


To assist in interpretation: 1) the coordinate system of Fig 1 is rotated 45° so that Δ*E* and Δ*T* are the horizontal and vertical axes, respectively; 2) the transitions are intentionally depicted as having the same initial and final values for Δ*T*. For the states below the Δ*E* axis (*i.e.*, 10 and 1-4), progression from left to right corresponds to increasingly reduced supply-demand balance, while the progression from right to left corresponds to increasingly improved supply-demand balance for the states above the axis (*i.e.*, 5-9). Note that the units assigned to the axes are arbitrary.

The 4→9 (red) transition in Fig F is associated with a change in Δ*E* (specifically, a fall in Δ*D* and increase in Δ*O*) larger than the increase in Δ*T*. A redistribution of Δ*D* and Δ*O* of the indicated magnitude is not easily accounted for solely by the washing out deoxyhemoglobin by fresh, (almost) fully oxygenated blood. A more plausible interpretation is a reduction in demand for O_2_ (such as could result from the inhibitory action of hyperemia-dependent nitric oxide production on cytochrome oxidase) concomitant with the hyperemia.

The 10→5 (blue) transition also shows a change in Δ*E* larger than that in Δ*T*. In addition, in this case the observed Δ*E* change (*i.e.*, a fall in Δ*D* and increase in Δ*O*) is in the opposite direction to what would result from an inrush of oxygenated blood in the absence of concomitant change in demand for O_2_. In this instance, a physiologically straightforward interpretation is an increase in O_2_ demand, by an amount greater than the increase the O_2_ supply can satisfy.

The 1→6 and 2→7 (green) transitions are hyperemia that, compared to the 10→5 case, is closer to being in balance with the change in O_2_ demand. The increase in Δ*D* and decrease in Δ*S* indicate that there still is net insufficient O_2_ supply but, in contrast to 10→5, this is accompanied by an increase in Δ*O*. Finally, the 3→8 (purple) transition is a hyperemic response that constitutes a close to exact match between increased O_2_ demand and its supply, where a large (but “just right”) increase in Δ*O* is accompanied by substantially smaller net increases in Δ*D* and Δ*S*.

The preceding conclusions regarding the (im)balance between demand for and supply of O_2_ are plausibility arguments, not deterministic ones. That is, the suggested interpretations represent our understandings of the conditions most likely to prevail in the resting state. Potential errors in these are likely to have little impact on the practical value of the outlined classification scheme, as they are not a criterion for achieving disease discrimination.
