# Supplementary material for: Hemoglobin state-flux: A finite-state model representation of the hemoglobin signal for evaluation of the resting state and the influence of disease
Source: PLoS One. 2018 Jun 8;13(6):e0198210. doi: 10.1371/journal.pone.0198210 (PMC5993307; doi:10.1371/journal.pone.0198210)
Supplement: S5 Appendix — Replots of transition intrinsic-flux measures, to highlight trends that are otherwise difficult to appreciate [includes Supporting Information Figs G and H]. (DOCX) [file pone.0198210.s005.docx]

**Informative Trends Among Hb-Component Coefficient Values**

Useful trends embedded in high-dimensional data are often difficult to appreciate. In Fig G, trends in mean flux amplitudes (indicated by the height of the bar graphs) among the Hb-components in affected breasts that constitute the different Hb-states are shown. As in previously represented plots, the column index encodes the state that the transitions proceed from and the row index indicates the state that is transitioned into. Inspection reveals that the amplitude for each component smoothly varies when values are compared that correspond to a clockwise or counter-clockwise progression among the states identified in Fig 1. Also seen is that the maximum amplitude that a given component has is associated with preferred states, as would be expected from the orientation of the various axes to each other.

**Fig G. Intrinsic transition-flux data for the affected breasts of the breast-cancer subject group.**


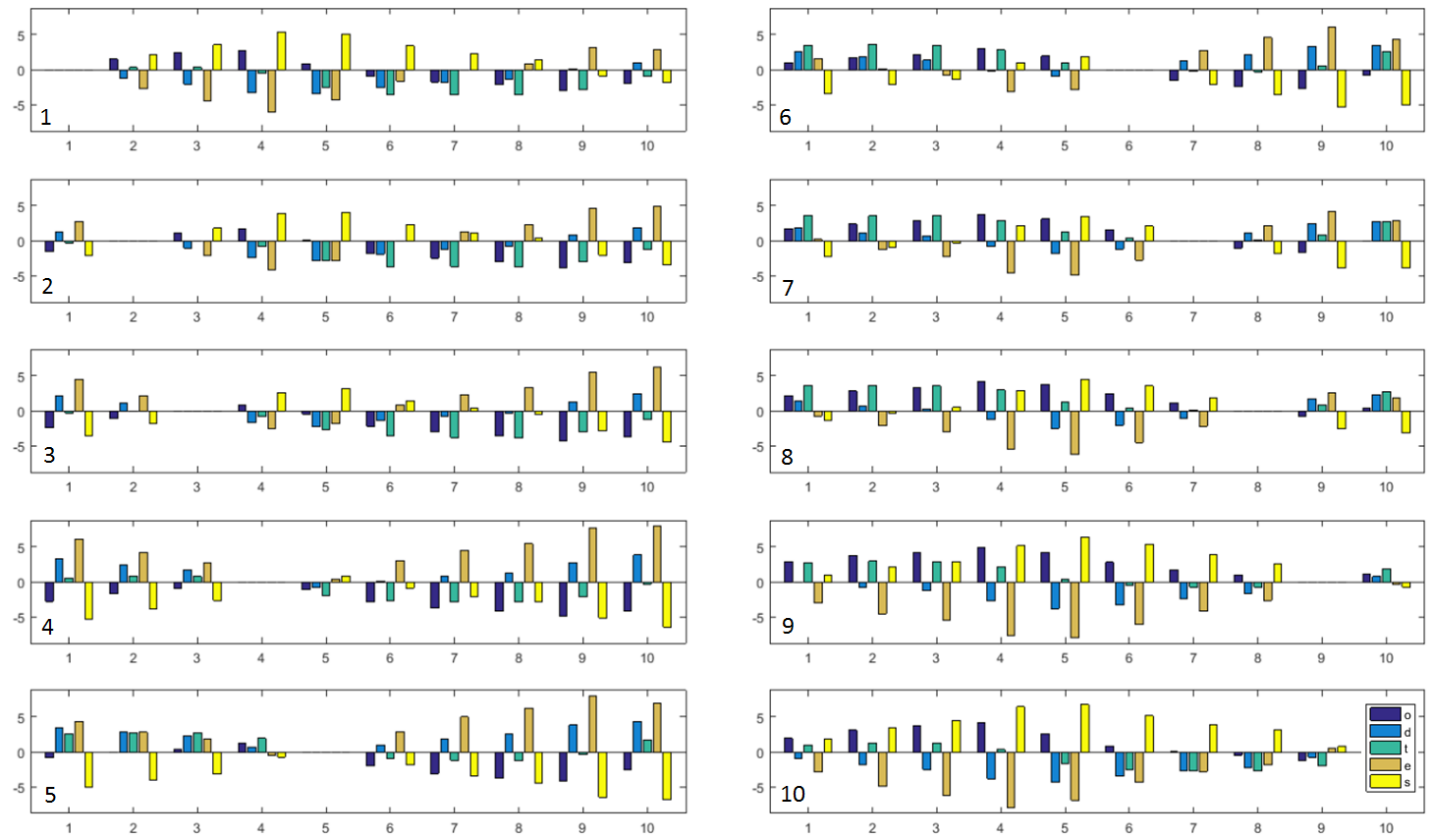


Plotted data (bar heights) are computed using Eq. (6), and are formatted as bar graphs to facilitate quantitative comparison. A common vertical scale (dimensionless) is achieved by multiplying the ΔHbO_2_Sat flux by 10^2^, and the others by 10^8^. Each panel corresponds to one row (explicitly identified by a number in the lower left corner) from the set of five maps analogous to those in Fig 9 (control group). Legend: ‘o’ = ΔoxyHb, ‘d’ = ΔdeoxyHb, ‘t’ = ΔtotalHb, ‘e’ = ΔHbO_2_Exc, ‘s’ = ΔHbO_2_Sat.

What is difficult to discern are the results shown in Figure H, where the associated flux amplitudes of individual Hb-components graphed in Fig G have been paired as a function of transition states. Here we have paired the ΔdeoxyHb and ΔoxyHb values for each state for the group comprising affected and contralateral unaffected breasts. While the equivalent information is also available from the flux values shown in Fig 9, explicit appreciation requires two transformation steps; 1) a pairing of the transition-dependent flux amplitudes for ΔdeoxyHb and ΔoxyHb values, and 2) plotting of each column of this paired data as a function. That the result is the noted ellipsoidal patterns seen is not apparent from an inspection of data represented in either Fig 9 or Fig G. Also evident is that the amplitude and orientation of the flux-transition ellipses differs significantly among the affected and unaffected breasts. In contrast to the ill-defined clouds of points in Fig 3, it appears that useful quantitation of the data represented in Fig H is likely available from comparisons of the major and minor axis lengths, eccentricity, angle of rotation with respect to the coordinate axes, and goodness of elliptical fit, among other easily discerned features.

**Fig H. Intrinsic fluxes and average difference between post- and pre-transition ΔoxyHb and ΔoxyHb.**


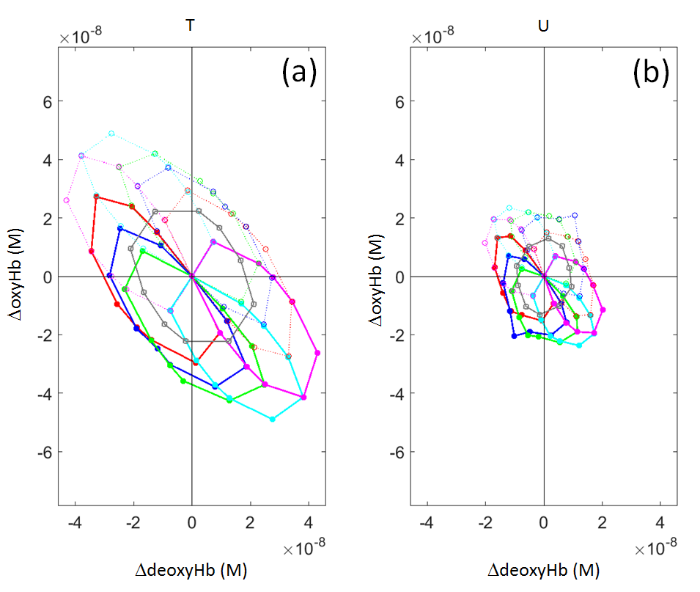


Colored curves: Plots of ΔoxyHb intrinsic flux vs. ΔdeoxyHb intrinsic flux, for the (a) tumor-bearing and (b) unaffected breast of the breast-cancer subjects. Each closed curve contains data for all transitions into one of the ten Hb states. Thus the red curve identifies transitions into State 1 from all other states. Successive transitions into the common post-transition state are identified by proceeding from the origin in the counter-clockwise direction. For the solid curves and symbols: red = transitions into State 1, dark blue = State 2, green = State 3, light blue = State 4, magenta = State 5. For the dotted curves with open symbols: red = transitions into State 6, dark blue = State 7, green = State 8, light blue = State 9, magenta = State 10. Gray curves: Average difference between post- and pre-transition ΔoxyHb vs. average difference between post- and pre-transition ΔdeoxyHb, for each of the ten states.
